# Early warning of regime switching in a complex financial system from a spillover network dynamic perspective

**DOI:** 10.1016/j.isci.2025.111924

**Published:** 2025-01-30

**Authors:** Sufang An, Xiangyun Gao, Feng An, Tao Wu

**Affiliations:** 1 School of Management, Hebei GEO University, Shijiazhuang 050031, China; 2 School of Economics and Management, China University of Geosciences, Beijing 100083, China; 3 School of Economics and Management, Beijing University of Chemical Technology, Beijing 100029, China; 4 College of Management Science, Chengdu University of Technology, Chengdu 610059, China; 5 Key Laboratory of Carrying Capacity Assessment for Resource and Environment, Ministry of Natural Resources, Beijing 100083, China; 6 Natural Resource Asset Capital Research Center, Hebei GEO University, Shijiazhuang 050031, China; 7 International Research Center for Neurointelligence, University of Tokyo, Tokyo 113-0033, Japan

**Keywords:** Social sciences, Business, Research methodology social sciences

## Abstract

Early warning of regime switching in a complex financial system is a critical and challenging issue in risk management. Previous research has examined regime switching through analyzing the fluctuation features in a single point in time series; however, it has rarely examined the dynamic spillovers across multivariable time series. This paper develops an early warning model of regime switching that incorporates a spillover network model and a machine learning model. Typical energy prices and stock market indices are selected as the sample data. The key spillover networks can be detected according to the distribution of the network indicators. The early warning signals can be captured by six typical machine learning models, and the random forest model has better performance. The robustness of the model is also discussed. Our study enriches regime switching research and provides important early warning signals for policymakers and market investors.

## Highlights

• Early warning of regime switching in a complex financial system is investigated
• A spillover network-based a machine learning model is established
• Early warning of regime switching with early warning days can be identified
• Random forest model shows higher performance

## Graphical abstract

**Figure:**
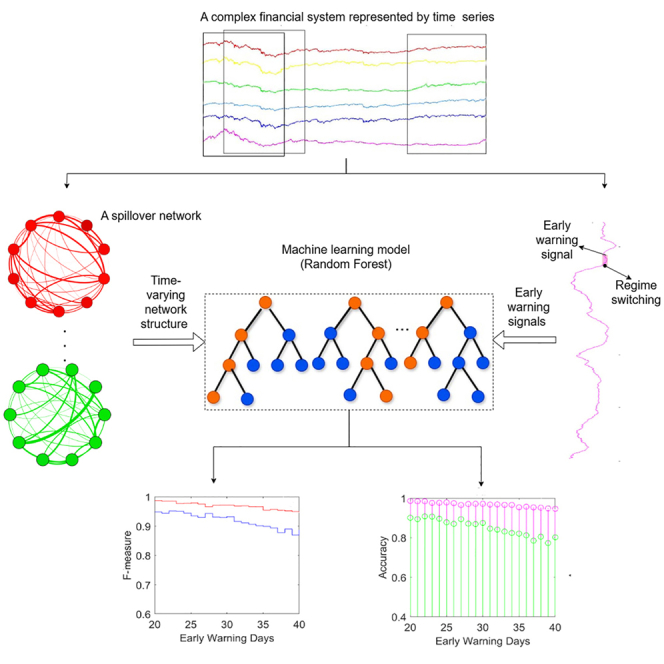

## Introduction

Early warning of regime switching is a critical and challenging issue in complex dynamical systems ranging from natural systems^1^^,^^2^^,^^3^^,^^4^^,^^5^ to social systems.^6^^,^^7^^,^^8^^,^^9^ The regime switching or regime shift indicates that the system changes its states abruptly.^1^^,^^7^^,^^10^^,^^11^^,^^12^ These sudden changes are often caused by gradual changes in internal properties or stressors, such as unpredictable exogenous drivers of change.^1^ Various early warning models (i.e., body size shifts as an early warning of whale population collapse^2^ and patterns in electroencephalogram (EEG) signals during preictal periods to predict epileptic seizures in medicine^5^) have been proposed for anticipating regime switching in a system,^1^^,^^7^ which can reveal the underlying mechanism and features of the complex dynamic system. In a financial system, the switch often alters the function of the system, reflecting that the changed regime may persist for many periods.^6^ The features or mechanisms of the system can be extracted as the early warning signals. For example, the subprime mortgage crisis in the United States started in 2007 not only penetrated the real economy but also spread throughout the world. At the beginning of the crisis, the mean and volatility in the stock return series changed abruptly and persisted for many periods. This finding indicates that features of changeable mean and volatility can be used as early warning signals to predict regime switching, which can reduce economic loss and even prevent systematic financial market collapse.^7^

Several models for investigating early warnings of regime switching in a financial system represented by time series have been established. These studies can be categorized into three groups. The first method is based on the regime-switching models in financial modeling.^13^^,^^14^^,^^15^ The time series can be characterized as fluctuations between recurring and different regimes, and early warnings of regime switching can be explored. The second method is based on the signaling approach^7^^,^^8^^,^^9^^,^^16^ in which potential early warning indicators, such as critical slowing down, flickering, and skewness, can be investigated. The third method is based on machine learning models,^17^^,^^18^^,^^19^^,^^20^^,^^21^ which are relatively new for forecasting early warnings of regime switching. Early warning models have already demonstrated great potential in the financial market, in which the fluctuation features of a single point in a time series have been focused on.^22^ However, there are several difficulties in early warning models. First, the state of the financial system may undergo a small change before reaching regime switching,^7^ indicating that a large change in the fluctuation features of a single point in a time series may be difficult to extract. Second, a real financial system often consists of many time series with interactions; for example, on April 20, 2020 (local time), the West Texas Intermediate (WTI) crude oil futures price plummeted to −37.63 dollars per barrel, but the crude oil system, which consists of several oil prices, did not collapse. In addition, the financial system’s regime is influenced by typical financial or global events, which affect information transmission across markets,^23^ which is referred to as volatility spillover. Several studies related to dynamic spillovers^24^^,^^25^^,^^26^^,^^27^ have demonstrated that the spillover structure across financial markets changes before and after these events. Above all, dynamic spillovers across multivariable time series can provide much more information than a single point’s fluctuation in a time series. Before reaching regime switching, the changeable fluctuation of a single point is small, but the spillovers during a short-term period represent a dynamic process. Therefore, it is necessary to understanding early warning signals in this regard.

In this work, we develop a hybrid model for early warning signals of regime switching in a complex financial system based on a spillover model combined with complex network theory^28^^,^^29^^,^^30^^,^^31^ and a machine learning model, namely, the spillover network-machine learning (SN-ML) model. Our proposed model can capture early warning signals of regime switching in a financial system with multivariable time series. The main contribution is that we investigate the dynamic structure of spillovers across multivariable time series instead of the fluctuation features of a single point in a time series to explore early warning of regime switching, and we establish a machine learning model to capture the key dynamic structure. Empirical financial data and six machine learning models can be used to test the model’s performance. A robustness analysis is also discussed.

## Results

### Dataset

To test the performance of our proposed model, this paper selects the energy and stock markets as the sample data.^26^^,^^27^^,^^32^ The Brent and WTI crude oil futures prices and natural gas futures prices are selected since they represent the major energy markets in the world, which are important industrial inputs. The seven main stock market indices, such as the Shanghai Stock Exchange (SSE), Financial Times Stock Exchange 100 Index (FTSE 100), Deutscher Aktienindex (DAX), Nikkei 225, S&P/asx200 Index (SP Index), Singapore Straits Times Index (STI), and S&P 500, can be selected. These indices include the major developed stock markets and China’s stock markets, which became the second-largest in the world in 2015.^32^ This paper selects more than ten years of daily data from January, 4, 2007 to April, 17, 2020 as the research target. Daily data are more appropriate for our research aim of capturing the dynamic structure of spillovers across financial markets. It is more detailed in its description of changeable spillover structures than weekly or monthly datasets are. In particular, market investors require analysis at the daily level to identify early warning signals in regime switching. Note that the missing data are deleted. All the data are downloaded from the websites and database can be given in the key resources table.

### Detection of regime switching

**Figure 1:**
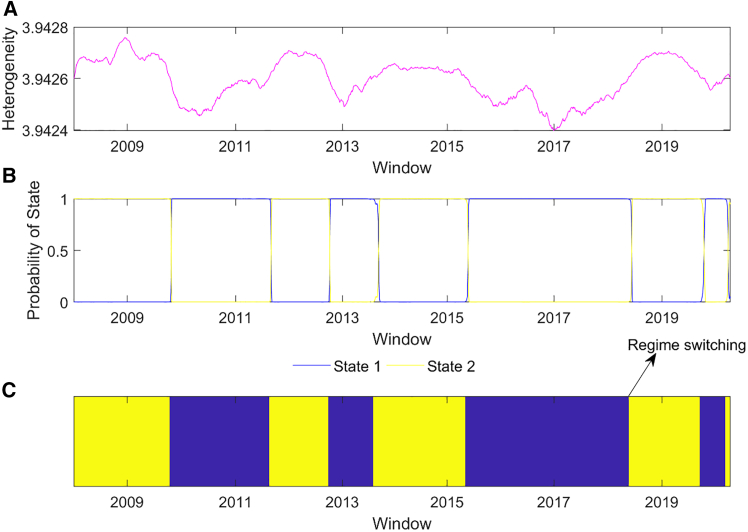
Detection of regime switching in a complex financial system (A) Spatial global residual entropy of the windowed time series. (B) Probability of two states. The window is the last day of the sub time series in the windowed time series. (C) The states of the windowed time series. The yellow and blue parts reflect the high and low states, respectively. The regime switching represents the switch between the high state and the low state.

We detect the regime switching of the heterogeneity in financial time series by proposing a model that combines an entropy model and a hidden Markov model (HMM). First, a windowed time series can be obtained by mapping an N-dimensional financial time series into many sub-time series evolving into each other. Second, after spatial global residual entropy has been determined to evaluate the heterogeneity of a windowed time series, an HMM with two states is established to detect the regime switching of heterogeneity in financial time series. Figure 1A illustrates the spatial global residual entropy, reflecting that the heterogeneity of financial time series is time-varying. Since complex events such as typical financial, econometric, and geopolitical events have a great influence on financial markets,^33^^,^^34^ we take the financial time series as a whole to compute their heterogeneity. Note that the model is related to the window width and step. We selected 220 days as the window width in this model because it spans approximately one year excluding weekends and holidays, and the step is one that includes the maximum overlapping information between adjacent windows. An HMM with two states is established to detect regime switching.

Figure 1B shows the probabilities of two states, indicating that the data’s heterogeneity belongs to one state with a high probability. The switch between the two states is given in Figure 1C. A high state suggests greater heterogeneity in the data, and a low state reflects lower heterogeneity in the data. These results indicate that there are numerous switches between the high state and the low state. Beginning in a high state, the system sustained this value until October 2009, indicating that the financial system experienced greater heterogeneity during the global financial crisis (2007–2009). This crisis caused sharp price volatility in financial markets. For example, the Brent oil price dropped sharply to less than $40 per barrel in the second half of 2008, which was an approximately 75% reduction in half a year. There was a switch from the high state to the low state, after which the financial system remained in a low state until August 2011. This suggests that the system has lower heterogeneity. The state jumps from low to high, and the state of the system was high until September 2012. This indicates that the system has greater heterogeneity. Compared with the state of the system during the global financial crisis, this indicates that the period of the system’s high state has become shorter. During these periods, price volatility in financial markets is mainly influenced by aggressive government policies that can stimulate the economy and other complex factors. For instance, countries such as Greece and Ireland were unable to help their overindebted financial institutions. At the same time, politics were turbulent in the Middle East and North Africa, especially the Syrian War in 2011, resulting in sharp cuts in oil production, which impacted Brent oil prices by 20%.^33^^,^^34^

In 2013, the system exhibited a low state over a short period of time. A switch from a low to high state occurred in August 2013. In April 2015, the state moved from high to low. This result reflects the greater heterogeneity of the financial system in 2014. In the second half of 2014, the decrease in oil prices was mainly due to the shale oil revolution in the United States. Subsequently, the system reached a low state in May 2018. In September 2019, the system reached a low state, and it reached a high state in March 2020. This indicates that the system shifted from a low to a high state during China-US trade frictions (2018–2019). This suggests that the system exhibited greater heterogeneity during this period. This shows that the speed of the switches between states increases, indicating the increasing uncertainty of the system.

### Time-varying structure of spillover networks

**Figure 2:**
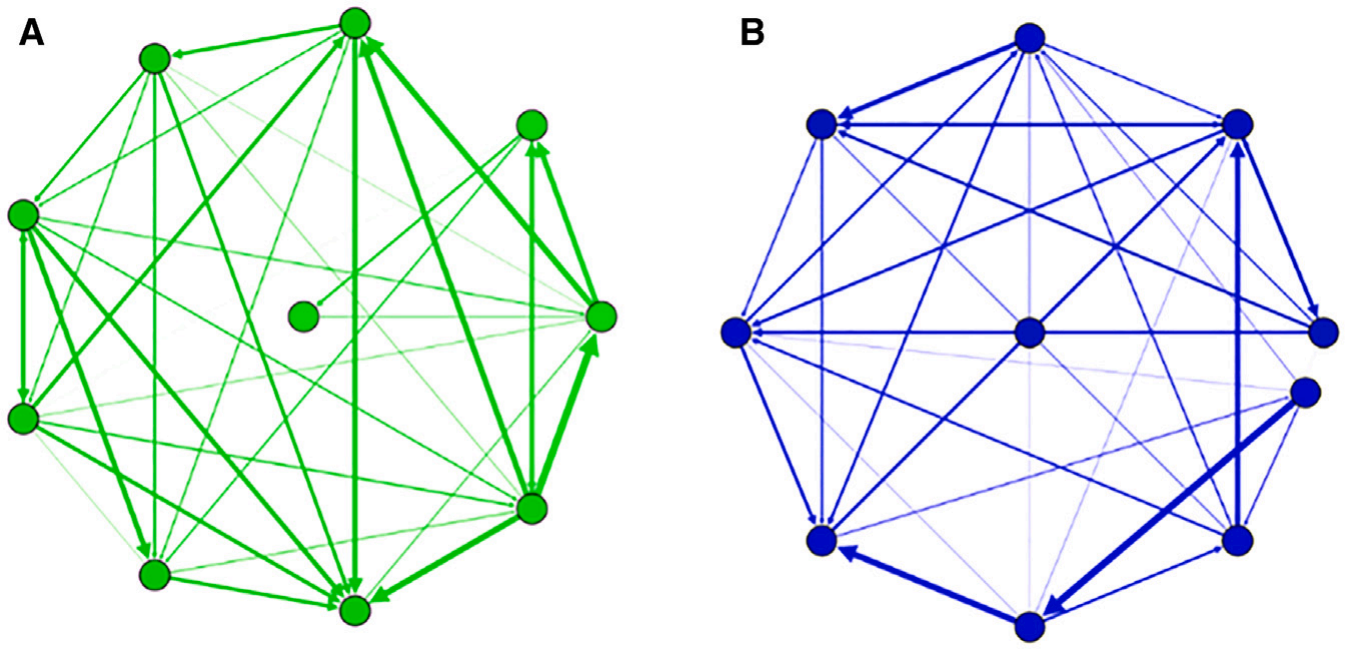
An example of spillover networks The node in a subfigure is a financial time series, and the weighted edge denotes the direction and magnitude of spillover flow between two time series. (A) Spillover network during the global financial crisis. (B) Spillover network during the COVID-19 pandemic.

Dynamic spillover networks are reconstructed from a windowed time series to explore the time-varying structure of spillovers among time series. A spillover network can be obtained from a sub-time series. The node of a spillover network is a financial time series, and the weighted edge denotes the direction and magnitude of spillover flow from one time series to another during the current short-term period. Based on the spillover network method, we obtain a sequence of spillover networks from a windowed time series. The sequence includes thousands of spillover networks that form an evolutionary process. Figure 2 presents an example of spillover networks during two different short-term periods. Subfigures A and B reflect a spillover network during the global financial crisis and the COVID-19 pandemic, respectively. The results indicate that the topological structures of spillover networks across periods are different.

**Figure 3:**
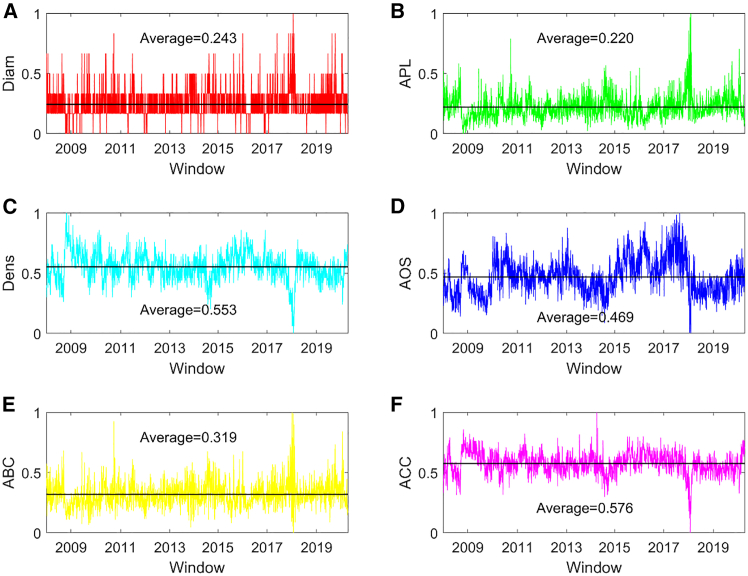
Dynamic topological structure of the spillover network The results are normalized network indicators (A) Dynamic diameter of the spillover network. Diam represents diameter (B) Dynamic APL of the spillover network. APL represents average path length. (C) Dynamic density of the spillover network. Dens represents density. (D) Dynamic AOS of the spillover network. AOS represents average out-strength. (E) Dynamic ABC of the spillover network. ABC represents average betweenness centrality. (F) Dynamic ACC of the spillover network. ACC represents average closeness centrality.

**Figure 4:**
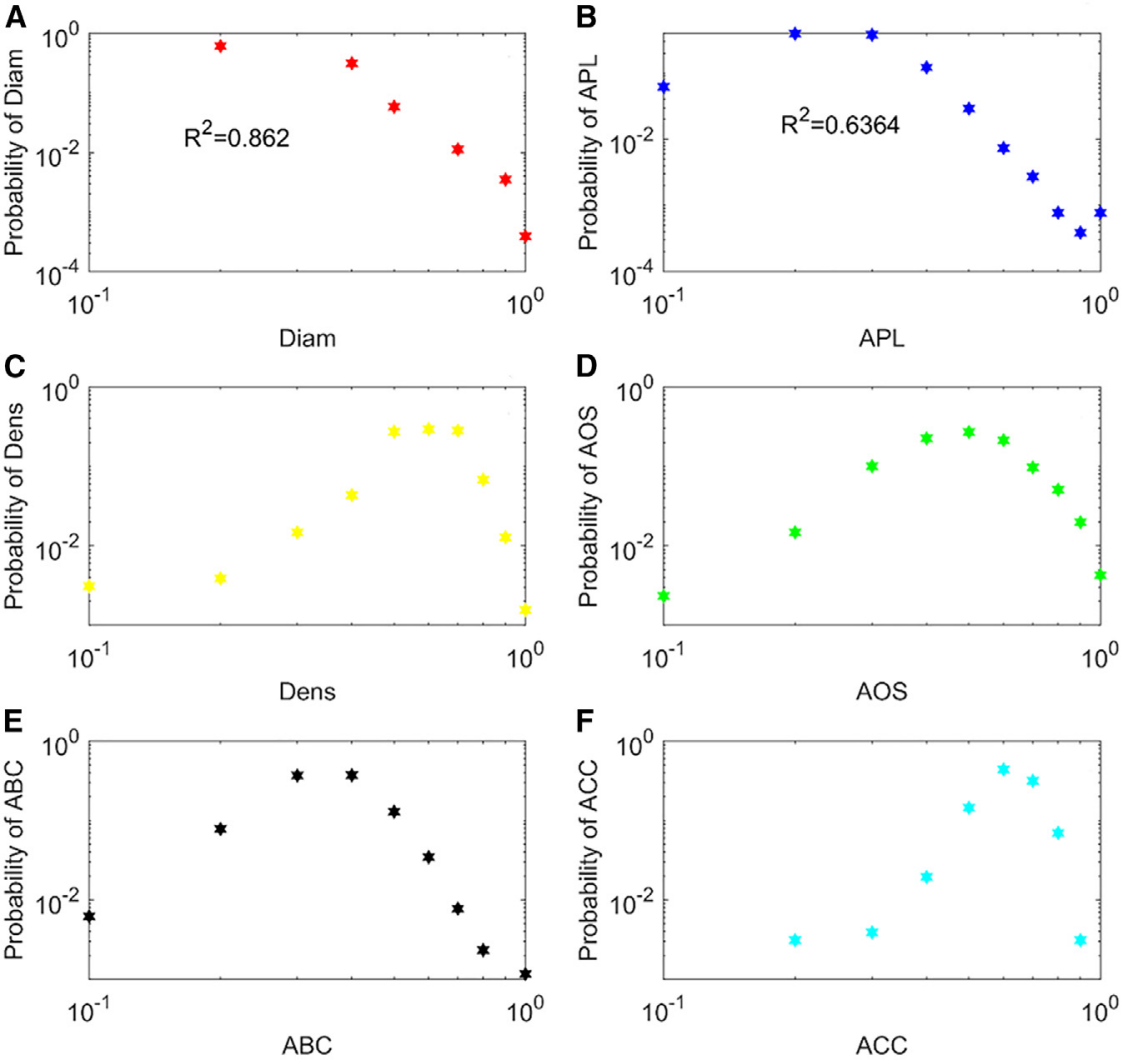
Distribution of the network indicators We define 0.1 as the interval extension of each network indicator to allocate the indicator into different intervals to analyze the relationship between the network indicator and its probability. The results are normalized network indicators. (A) Distribution of diameter. Diam represents diameter. (B) Distribution of APL. APL represents average path length. (C) Distribution of density. Dens represents density. (D) Distribution of AOS. AOS represents average out-strength. (E) Distribution of ABC. ABC represents average betweenness centrality. (F) Distribution of ACC. ACC represents average closeness centrality.

This study investigates the dynamic network structure of spillovers across stock and energy time series as a whole when typical financial or economic events occur, such as the global financial crisis and the COVID-19 pandemic. Figure 3 illustrates the dynamic network indicators of spillovers across the stock and energy time series. The distributions of these indicators are shown in Figure 4, which can be used to understand the characteristics of spillover networks throughout the entire sample period. Figure 3A presents the normalized diameter of a spillover network, indicating that the linear size of a network changes with time in different windows. The average value is 0.243. The maximum value of these normalized values occurred in January 2018. Our findings indicate that it fluctuates around the average value during the entire sample period. Furthermore, the normalized diameter in most spillover networks during the global financial crisis was larger than the average value, and it had a lower value in most networks during the COVID-19 pandemic. In addition, the normalized diameter follows a power-law distribution, as shown in Figure 4A, reflecting that most networks have smaller diameters.

The normalized average path length is illustrated in Figure 3B, which indicates that the transmission performance among spillover flows is time varying. The average value is 0.220. The maximum value of these indicators occurred in February 2018, and the minimum value occurred in October 2008. Beginning with a higher value, the normalized average path length in most networks was greater than the average value until the end of 2008 and then smaller than the average value until the beginning of 2010. In the early and late periods of China-US trade frictions, the indicator had higher values. When the COVID-19 pandemic occurred, it trended from a higher value to a lower value. In addition, the normalized average path length follows a power-law distribution, as shown in Figure 4B. This indicates that most networks have lower transmission performance.

The normalized network density is shown in Figure 3C, which reflects that a ratio related to the number of edges changes with time. The average value is 0.553. The maximum value of these indicators occurred in October 2008, and the minimum value occurred in January 2018. Furthermore, during the global financial crisis, the density in most networks decreased until the end of 2008 and then increased until the end of 2009. In the early and late periods of China-US trade frictions, the indicator had a lower value, but it increased when the COVID-19 pandemic occurred. Additionally, its distribution is presented in Figure 4C, and the key spillover networks with the highest probability of density can be detected.

The normalized average out-strength of a spillover network is presented in Figure 3D, indicating that the average importance of spillover flows in a network changes with time. The average value is 0.469. The maximum value occurred in August 2017 and the minimum value occurred in February 2018. Furthermore, during the global financial crisis, the average out-strength in most networks decreased, indicating that the average importance of spillover flows was smaller. In most networks, the network indicator had a greater value from 2015 to 2017. It was lower during the period of China-US trade frictions. When the COVID-19 pandemic occurred, it decreased from a lower value to a higher value. In addition, its distribution is shown in Figure 4D, and the key spillover network with the highest probability of average out-strength can be identified.

Figure 3E represents the normalized average betweenness centrality of a spillover network, reflecting that the average importance of nodes as bridges in a network is time varying. The average value is 0.319. The maximum value occurred in December 2017, and the minimum value occurred in January 2018. Furthermore, the normalized average betweenness centrality in most networks was greater until the end of 2008, and then it was lower until the end of 2009. In the early and late periods of China-US trade frictions, the indicator had a larger value. When the COVID-19 pandemic occurred, it had a lower value. In addition, the key network with the highest probability of average betweenness centrality can be detected according to its distribution, as shown in Figure 4E. Figure 3F presents the normalized average closeness centrality of a spillover network. The average value is 0.576. The maximum value was in April 2014, and the minimum value was in January 2018. Furthermore, during the global financial crisis, the normalized average closeness centrality in most networks had a smaller value until the end of 2008 and then had a larger value until the end of 2009. The indicator in most networks had a lower value during the period of China-US trade frictions. When the COVID-19 pandemic occurred, it has greater value. In addition, the key network with the highest probability of average closeness centrality can be identified according to its distribution, as shown in Figure 4F.

### Early warning signals via machine learning

#### Model performance

An early warning model of regime switching via a machine learning model is established. The main process is described as follows. (1) We can obtain the sequence of early warning signals according to the definition of the early warning signal of regime switching. We then construct a database consisting of the output variable and the input variable, where the output variable is the early warning signal of regime switching, and the input variable is the time-varying structure of spillover networks. (2) We randomly divide the database into a training set and a testing set. The training set comprises 80% of the database, whereas the testing set comprises the remaining 20%. The training set can be used to train the model based on a machine learning model, whereas the testing set can be used to evaluate the model performance. In addition, compared with the whole dataset, the samples of early warning signals belong to a minority, which leads to a large bias toward the majority data in the data training process. The cross-sectional model can be used to preprocess the training data^35^ due to the imbalance of the output variables. (3) Six typical machine learning models, including support vector machine (SVM),^36^^,^^37^ gradient-boosted decision tree (GBDT),^38^ artificial neural network (ANN),^39^ deep neural network (DNN),^40^ random forest (RF),^41^ and k-nearest neighbors (KNNs),^42^ are selected to solve our early warning model; the F-measure and accuracy are used to measure the model performance. The indicator of our model depends not only on the reliability of the dataset with early warning days but also on the selected machine learning model.

**Figure 5:**
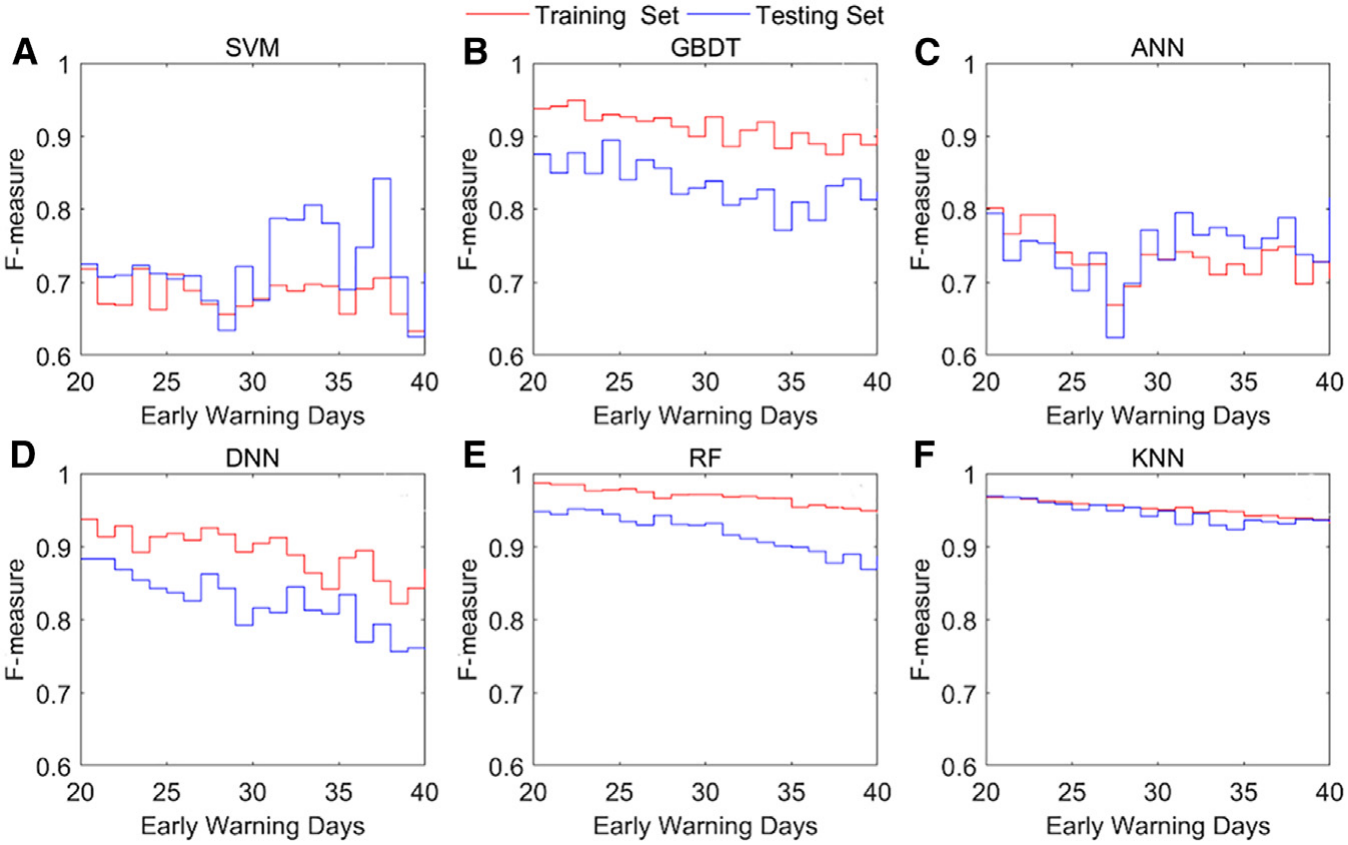
Dynamic F-measures of the training set and testing set in the machine learning models (A) Dynamic F-measures of the training set and testing set in the SVM. (B) Dynamic F-measures of the training set and testing set in the GBDT. (C) Dynamic F-measures of the training set and testing set in the ANN. (D) Dynamic F-measures of the training set and testing set in the DNN. (E) Dynamic F-measures of the training set and testing set in the RF. (F) Dynamic F-measures of the training set and testing set in the KNN.

The F-measures of the six machine learning models with different early warning days are illustrated in Figure 5. The results show that the F-measures of the training and testing sets on different early warning days in a model are time-varying. Table 1 shows the maximum, minimum, and average F-measures of the training and testing sets. For example, Figure 5B illustrates the dynamic F-measures of the GBDT for the training and testing sets. The dynamic F-measure of the GBDT for the training set ranges from 0.875 to 0.949, with an average value of 0.912. The mean F-measure of the GBDT on the testing set is 0.835, and the range of the F-measure is between 0.771 and 0.894. As the number of early warning days increases, the F-measures of a machine learning model fluctuate. Users can select early warning days based on the economic cycle and the actual situation. Additionally, our findings indicate that the RF and KNN models have greater F-measures than the other models do when comparing the ranges of the F-measures and their means for the six models.

**Table 1:** F-measures of the training set and testing set

Model	Indicator	F-measure of training set	F-measure of testing set
SVM	Max.	0.719	0.842
	Min.	0.633	0.625
	Average	0.680	0.723
GBDT	Max.	0.949	0.894
	Min.	0.875	0.771
	Average	0.912	0.835
ANN	Max.	0.801	0.817
	Min.	0.669	0.624
	Average	0.734	0.747
DNN	Max.	0.938	0.883
	Min.	0.822	0.757
	Average	0.892	0.822
RF	Max.	0.987	0.951
	Min.	0.947	0.869
	Average	0.968	0.919
KNN	Max.	0.968	0.969
	Average	0.932	0.923
	Min	0.952	0.946

Min., minimum; Max., maximum.

**Figure 6:**
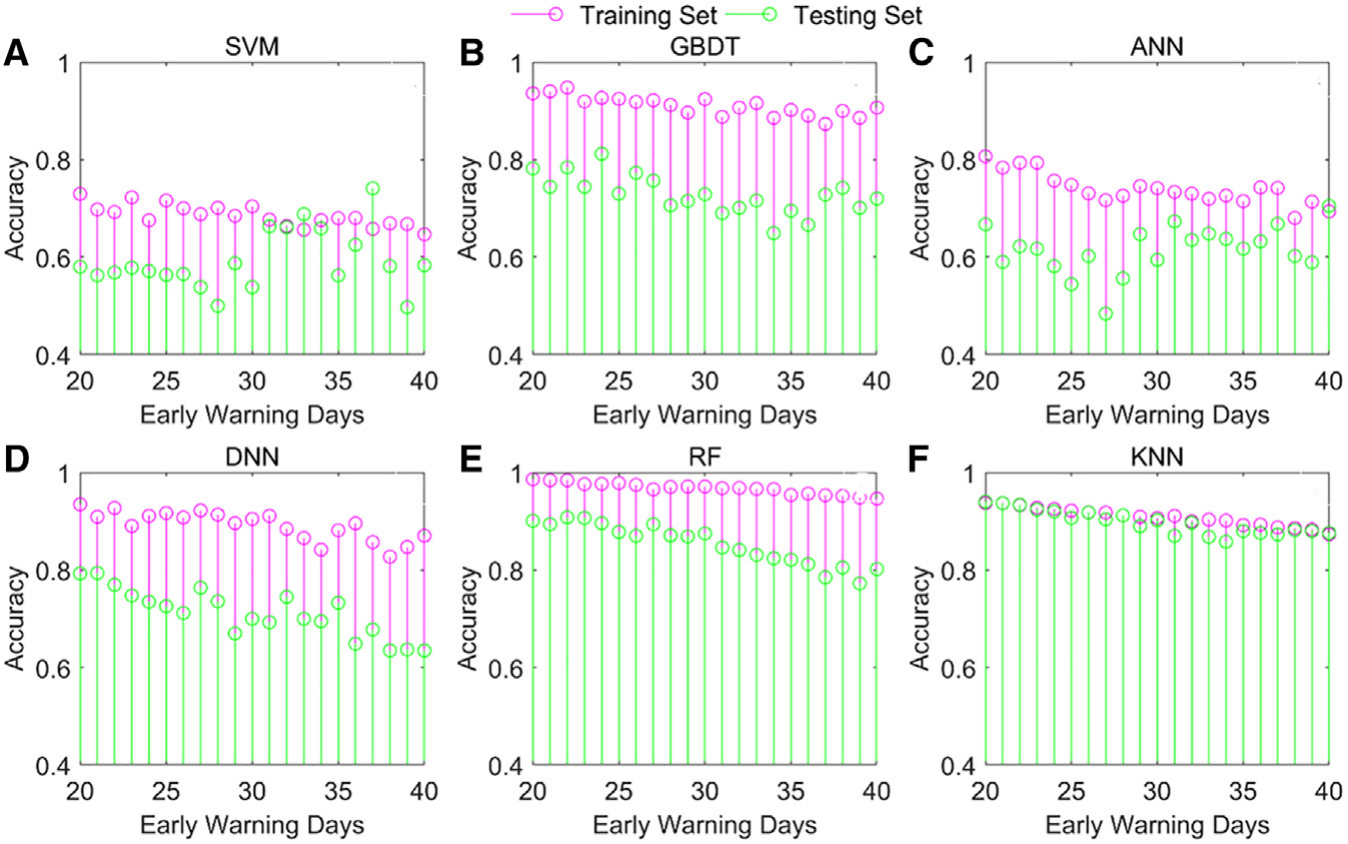
Dynamic accuracies of the training set and testing set in the machine learning models (A) Dynamic accuracies of the training set and testing set in the SVM. (B) Dynamic accuracies of the training set and testing set in the GBDT. (C) Dynamic accuracies of the training set and testing set in the ANN. (D) Dynamic accuracies of the training set and testing set in the DNN. (E) Dynamic accuracies of the training set and testing set in the RF. (F) Dynamic accuracies of the training set and testing set in the KNN.

Figure 6 shows the accuracies of the six machine learning models under different early warning days. These results show that the accuracies of the training and testing sets with early warning days in a machine learning model change with time. Table 2 reflects the maximum, minimum and average accuracies of the training and testing sets. For example, Figure 6D presents the dynamic accuracies of the training and testing sets in the DNN. The accuracy of the training set ranges from 0.827 to 0.935 and its mean value is 0.891. The accuracy of the testing set is between 0.635 and 0.794, and the average value is 0.712. These findings indicate that the RF and KNN models have greater accuracies on the training and testing sets when comparing the ranges of the accuracies and their means for the six models.

**Table 2:** Accuracies of the training set and testing set

Model	Indicator	Accuracy of training set	Accuracy of testing set
SVM	Max.	0.730	0.741
	Min.	0.647	0.497
	Average	0.685	0.591
GBDT	Max.	0.948	0.812
	Min.	0.873	0.649
	Average	0.911	0.728
ANN	Max.	0.807	0.705
	Min.	0.680	0.484
	Average	0.740	0.615
DNN	Max.	0.935	0.794
	Min.	0.827	0.635
	Average	0.891	0.712
	Max.	0.986	0.908
RF	Min.	0.947	0.773
	Average	0.968	0.853
	Max.	0.937	0.940
KNN	Min.	0.873	0.858
	Average	0.908	0.898

Min., minimum; Max., maximum.

Overall, the F-measures and accuracies of the training set and testing set are time-varying. The model performances of RF and KNN are better than those of the other models. This indicates that when we use RF and KNN models, there are few occasional false alarms in the models. RF is the best choice because it handles unbalanced classifications easily, performs well with high-dimensional databases, and can handle data in parallel.

#### Calculating the early warning signals

**Figure 7:**
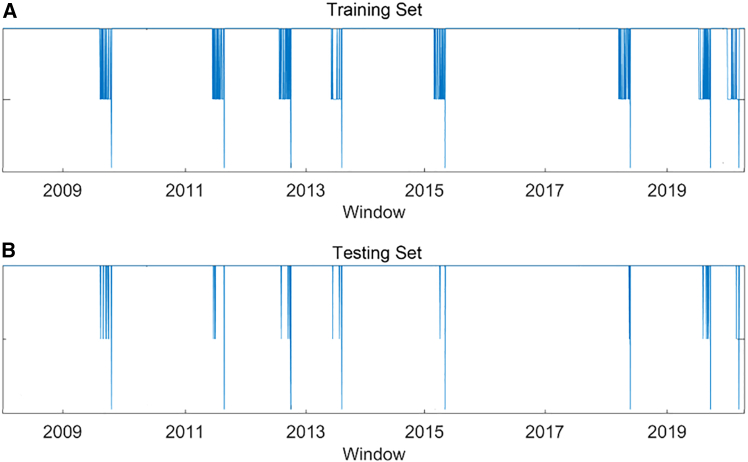
An example of the detection of early warning signals of regime switching The long line indicates regime switching, whereas the small line represents the early warning signals related to regime switching. (A) Early warning signals of regime switching on the training set. (B) Early warning signals of regime switching on the testing set.

The early warning signals of regime switching may be calculated after choosing the machine learning model and the early warning days. The DNN model and the early warning days of 40 days can be used as examples in this section. Figure 7 shows the early warning signals of the training set and testing set in the DNN, where the samples are correctly classified. The long line indicates regime switching, whereas the small line represents the early warning signals related to regime switching. For example, Figure 7B reflects early warning signals and the regime switching signals on the testing set. For instance, our proposed model can identify regime switching and several early warning signals between 2009 and 2011. This indicates that some early warning signals alert financial systems to regime switching within 40 days.

Above all, the results indicate that the time-varying structure of the spillovers across financial time series may provide some early warning signals of regime switching because of the analysis of model performance and the aforementioned example of early warning signals.

#### Robustness analysis

In this section, we discuss the robustness of our proposed model. First, we select a new window width of 200 days, as the window width to respond to approximately 11 trading months in the original daily database can be selected, and the step size can be set to one to ensure information with transmission and memory. All the figures in this section can be given in the supplemental information. Our findings indicate that the trend of the state under a window width of 200 is similar to our original results (Figure S2). The dynamic structure of spillover across financial markets is also time-varying (Figure S3). The result of the early warning model that is based on machine learning indicates that RF performs better and it is the recommended as the preferred machine learning model (Figures S4 and S5).

Second, we add the financial time series named US wheat and gold futures prices to the original daily database since time-varying spillovers exist between these series and some variables in our original database.^43^^,^^44^^,^^45^^,^^46^ The window width is 220 days, and the step size is one. The results indicate that the heterogeneity of the new daily database belongs to one state with a high probability (Figure S6). The dynamic structure of spillover across financial time series is also time-varying (Figure S7). Note that we randomly select 70% of the database as the training set and 30% of the database as the testing set to evaluate the early warning model performance. The results show that the RF performs better (Figures S8 and S9).

Third, we select the weekly database to discuss the robustness of our proposed model since the differences in closing times among some national equity markets should be considered when the lead-lag relationships across country returns in the daily database are analyzed.^47^ The selected variables and time period of the sample data are consistent with those in the original daily database. The window width is 220 days, and the step size is one day. The results indicate that the heterogeneity of the weekly database belongs to one state with a higher probability (Figure S10). Both the diversity of switches (Figure S10) and the diversity of spillover structures (Figure S11) in the weekly database are less than those in the original daily database. This implies that the daily database offers a more detailed description of features in the time series. Note that we randomly select 70% (30%) of the database as the training (testing) set to evaluate the early warning model performance. The results show that the proposed model still performs well on a weekly database, and RF is the recommended choice owing to its performance and computing rate (Figures S12 and S13).

## Discussion

This study proposes a spillover network-based machine learning model for early warning of regime switching in a complex financial system with multivariable time series. We select typical energy futures prices and stock market indices as the sample data. First, regime switching of heterogeneity in financial time series can be detected based on the HMM and entropy theory. Our findings indicate that the financial system switch between different states faster after China-US trade frictions (2018–2019). Second, thousands of spillover networks across financial time series are established. Our findings suggest that the direction and magnitude of spillover flows change with time. The key spillover networks with the highest probability of network indicators can be detected according to their distribution. Third, an early warning model of regime switching based on time-varying spillovers is proposed, and a machine learning model, such as an SVM, GBDT, ANN, DNN, RF, or KNN, can be built to calculate it. The results indicate that the early warning model using RF has better model performance, and a robustness analysis is discussed.

This paper explores the dynamic structure of spillovers across multivariable time series to investigate early warning of regime switching in a financial system. Our model can capture some early warning signals to anticipate regime switching and can be applied to a broad range of financial markets, such as stock markets, energy markets, and commodities. Policymakers can gain a deeper understanding of the dynamic structure of spillovers among the energy and stock markets when typical financial and economic events occur. They can also pay more attention to early warning signals of regime switching to protect against contagion effects and foster market stability. Additionally, market investors can pay attention to the dynamic process of spillovers. They can employ dynamic graphical tools to monitor the time-varying structure of spillovers and detect early warning signals of regime switching. They should develop efficient hedge strategies to reduce investment losses.

### Limitations of the study

This study proposes an early warning model of regime switching in a complex financial system with multivariable time series based on a spillover network and a machine learning model. Although the model performs well, the results are limited by the construction of a spillover network across typical financial markets and the selection of machine learning models. In addition, the model ignores the time differences between financial markets in the daily database. In the future, other economic networks and advanced machine learning models, such as graph neural networks, can be used to improve the performance of early warning models.

## Resource availability

### Lead contact

Requests for further information or materials should be directed to the lead contact, Xiangyun Gao (gxy5669777@126.com).

### Materials availability

This study did not generate any new materials.

### Data and code availability

• Data in this paper can be downloaded from the websites and the database which are listed in the key resources table.
• The codes used for this paper are available from the lead contact upon request. And the main software and algorithms in our code are listed in the key resources table.
• Any additional information required to reanalyze the data reported in this paper is available from the lead contact upon request.

## Acknowledgments

This work was supported by the 10.13039/501100001809National Natural Science Foundation of China (nos. 42101300, 72371229, 71991485, 72401043, 71991481, and 71991480), the Basic Science Center Project of the National Natural Science Foundation of China (no. 72088101, the Theory and Application of Resource and Environment Management in the Digital Economy Era), the 10.13039/501100012226Fundamental Research Funds for the Central Universities (3-7-8-2023-01), and the PhD Research Startup Foundation of Hebei GEO University (BQ2024015).

## Author contributions

S.A., methodology, data curation, software, writing – original draft. F.A., conceptualization, methodology, validation. X.G., methodology, funding acquisition, project administration. T.W., writing – review and editing, visualization.

## Declaration of interests

The authors declare no competing interests.

## STAR★Methods

### Key resources table

**Table:** 

REAGENT or RESOURCE	SOURCE	IDENTIFIER
**Deposited data**		

Brent and WTI crude oil futures prices Natural gas futures prices	Energy Information Administration and investing company	http://www.eia.govhttps://www.investing.com
Seven main stock market indices, U.S. wheat and gold futures prices.	Investing company or Wind database	https://www.investing.com Wind database

**Software and algorithms**		

R 4.0	R Core Team	https://www.r-project.org/
SpatEntropy	R package	https://cran.r-project.org/web/packages/SpatEntropy/index.html
depmixS4	R package	https://cran.r-project.org/web/packages/depmixS4/index.html
mgarchBEKK	R package	https://cran.r-project.org/web/packages/mgarchBEKK/index.html
nnet	R package	https://cran.r-project.org/web/packages/nnet/index.html
H2O	R package	https://cran.r-project.org/web/packages/h2o/index.html
E1071	R package	https://cran.r-project.org/web/packages/e1071/index.html
randomForest	R package	https://cran.r-project.org/web/packages/randomForest/
ggplot2	R package	https://cran.r-project.org/web/packages/ggplot2/index.html
caret	R package	https://cran.r-project.org/web/packages/caret/index.html

### Experimental model and study participant details

There are no experimental model or study participants to include in this study.

### Method details

We propose the SN–ML model, an early warning model, as shown in Figure S1 in the supplemental information. The four steps in the model can be summarized as follows. First, a windowed time series W, including M sub-time series that evolve into each other, can be obtained by mapping an N-dimensional financial time series Y. Second, after spatial global residual entropy has been determined to evaluate the heterogeneity of a windowed time series W, we obtain a sequence of spatial global residual entropies ${\left(e\left(1\right),e\left(2\right),\ldots ,e\left(m\right)\right)}_{m=1}^{M}$. An HMM is established to detect the regime switching of heterogeneity in a time series. A sequence of early warning signals ${\left({SP}_{1},{SP}_{2},\ldots ,{SP}_{m}\right)}_{m=1}^{M}$ can be calculated by a relationship function that is related to regime switching. Third, dynamic spillover networks ${\left(G_{1},G_{2},\ldots ,G_{m}\right)}_{m=1}^{M}$ are reconstructed from a windowed time series W to explore the time-varying structure of spillovers among time series. Fourth, the time-varying structure of spillovers is the input data Index(W), and the early warning signal series is the output data SP. A machine learning model can be established to train the nonlinear relationship function between these two sets of data, where the function is Noli(Index(W))=SP. The performance of the proposed model is also discussed.

#### Constructing a windowed time series

When describing the dynamic process of time series in complex system theory,^48^ a rolling window is a general method that divides time series into numerous interconnected blocks. An N-dimensional financial time series with each component of length T is denoted as:

(Equation 1) $$Y={\left(y_{i}^{\left[1\right]},y_{i}^{\left[2\right]},\ldots ,y_{i}^{\left[N\right]}\right)}_{i=1}^{T}$$

where ${\left(y_{i}^{\left[n\right]}\right)}_{i=1}^{T}$ represents a component. We use a fixed-length rolling window to divide the holistic time series into M sub-time series, where the window slides with time from left to right in steps of τ, the window width is ω, and generally, ω>N. M=[(T−ω)/τ+1]. Suppose that $i_{m}=\tau \left(m-1\right)+1$, $T_{m}=\tau \left(m-1\right)+\omega$; then, the m th sub-time series with each component of length ω can be described as follows:

(Equation 2) $$W={\left({W_{1},W_{2},\ldots ,W}_{m}\right)}_{m=1}^{M}$$

(Equation 3) $$W_{m}={\left(y_{i}^{\left[1\right]},y_{i}^{\left[2\right]},\ldots ,y_{i}^{\left[N\right]}\right)}_{i=i_{m}}^{T_{m}}$$

Each of these sub-time series is made up of an ω×N data matrix, and they evolve into each other. These sub-time series can then be used to describe the time series’ dynamic process. These sub-time series are referred to as a windowed time series W. According to the economic cycle, the window width ω can be selected as one month, one year, or two years.^25^ If there is only one window, then the simplest model is when ω is equal to the length of the entire sample. Additionally, τ satisfies τ≤ω to maintain the data continuity.^48^ Each sub-time series has the characteristics of memory and transitivity when the step τ is equal to 1, which means that each sub-time series concludes the maximum overlapping information.^25^^,^^49^ The model is the highest-dimensional windowed time series.

#### Detection of regime switching

Entropy is a measure of heterogeneity in many applied research fields. To measure the effect of space on a variable, Altieri et al.^50^ proposed spatial entropy, which was first introduced in our study. Suppose a random variable X with I possible categories. The information function is denoted as $\left(p\left(x_{i}\;\right)\right)=$ $\log\left(1/p\left(x_{i}\right)\right)$, and Shannon’s entropy of variable X can be denoted as:

(Equation 4) $$H\left(X\right)=\sum_{i=1}^{I}p\left(x_{i}\;\right)\log\left(1/p\left(x_{i}\right)\right)$$

Suppose a transformed variable Z of the study variable, i.e., pairs of relations of the variable of interest, R=binom(n+1,2) represents the number of possible pairs of categories of variables, n is the number of observations, and $p\left(z_{r}\right)$ represents the probability of the *r*th pairs of realizations, which is estimated by its relative frequency. Shannon’s entropy of the Z variables can be denoted as:

(Equation 5) $$H\left(Z\right)=\sum p\left(z_{r}\right)\log\;\left(1/p\left(z_{r}\right)\right)$$

where the Shannon’s entropy of the Z variable ranges from 0 to log (R), indicating that it calculates all possible pairs in the observation area. Suppose that the random variable $w_{k}=\left[{d_{k-1},d}_{k}\right]$ indicates the interval of the distances. If a variable A is constructed by a variable Z and the conditional probability mass function $p_{Z|A}={\left(p\left(z_{1}\;|A\right),\ldots ,p\left(z_{R}\;|A\right)\right)}^{\prime }$, then H $\left(Z|A\right)=\sum p\left(z_{r}\;|A\right)\log\left(1/p\left(z_{r}\;|A\;\right)\right)$. $p\left(z_{r}\;|w_{k}\right)$ represents the probability of the *r*th pair of realizations, which is estimated by its relative frequency, and $p\left(z_{r}\;|w_{k}\;\right)$ is an element of $p\left(Z\;|w_{k}\;\right)$. The Shannon’s entropy of $H\left(Z\;|w_{k}\right)$ can be described as the spatial partial entropy:

(Equation 6) $$H\left(Z\;|w_{k}\right)=\sum p\left(z_{r}\;|w_{k}\;\right)\log\left(1/p\left(z_{r}\;|w_{k}\;\right)\right)$$

Since spatial partial entropy can be summarized to obtain a global measure based on probability $p\left(w_{k}\right)$, this measure is called spatial global residual entropy:

(Equation 7) $$H{\left(Z\right)}_{W}=\sum p\left(w_{k}\right)H\left(Z\;|w_{k}\right)$$

Therefore, spatial global residual entropy can estimate data heterogeneity from the role of space. When the distribution of the ω×N data matrix in two dimensions may be considered the spatial distribution of N classes of data, we transform the sub-time series $W_{m}$ into point data categorical marks in this study.^50^ To explain the heterogeneity of the sub-time series in a window, we then compute the spatial global residual entropy of the data points named e(m). The windowed time series W can be transformed into a sequence of spatial global residual entropies ${\left(e\left(1\right),e\left(2\right),\ldots ,e\left(m\right)\right)}_{m=1}^{M}$.

Second, according to Hamilton,^51^ regime switching is a significant issue in financial markets. The HMM is a general model used to detect regime switching.^52^^,^^53^ This model consists of several states, each of which has a certain probability of transiting into another state. We describe a discrete HMM, and some notations are denoted as follows:

$Q=\left\{q_{1},q_{2},\ldots ,q_{N}\right\}\text{:}$ Set of the N states

$A=\left\{a_{ij}\right\}$: Transition probability matrix, where $a_{ij}$ represents the transition probability from state $q_{i}$ to state $q_{j}$, $\sum_{j=1}^{N}a_{ij}=1$

$O=\left\{o_{1,}o_{2},\ldots {,o}_{T}\right\}$: a sequence of T observations

$B=\left\{b_{i}\left(o_{t}\right)\right\}$: a sequence of state observation likelihoods, where $b_{i}\left(o_{t}\right)$ is the state observation likelihood of observation $o_{t}$ at the current state $q_{i}$.

$\pi =\left\{\pi_{i}\right\}$: Initial probability distribution over states, where $\pi_{i}$ is the probability that the Markov chain will start state i and $\sum_{i=1}^{N}\pi_{i}=1$.

There are two assumptions. A state’s probability depends on the previous state, and the probability of an output observation $o_{t}$ depends on the state that produces the state $q_{i}$. The HMM can be characterized in three parts: (1) Given an HMM λ={A,B} and an observation sequence O, examine the likelihood P(O|λ). (2) Given an observation sequence O and an HMM λ={A,B}, investigate the best hidden state sequence Q. (3) Given an observation sequence O and the set of states in the HMM, study the HMM parameters AandB.

In this paper, we first measure the probability of the state based on the HMM and the dynamic heterogeneity of the windowed time series, where the state has two types. It is similar to the stock market, which can be a bear or bull market. Second, we select the state with the highest probability as the final state and then obtain a state sequence $RS=\left\{R_{m}\right\}$, where m=1,2,…,M. The element $R_{m}$ is denoted as:

(Equation 8) $$R_{m}=\left\{ \begin{array}{c} \text{Low},\text{state}\;\text{belongs}\;\text{to}\;\text{type}\;1\\ \text{High},\text{state}\;\text{belongs}\;\text{to}\;\text{type}\;2 \end{array}\right.$$

Furthermore, the state sequence can describe the dynamic process of the dynamic heterogeneity of time series. Regime switching occurs when the state in the sequence switches from one state to another.

#### Time-varying structure of spillover networks

Dynamic spillovers across financial markets have been investigated using a hybrid framework that incorporates an economic model and complex network theory. The primary framework maps the multivariable financial time series into a sequence of spillover networks that evolve into one another over time. Subsequently, the dynamic topological structure of the spillover networks is analyzed during different time periods. A number of studies have already demonstrated the great potential of exploring dynamic spillovers across financial markets from a variety of perspectives, including time,^24^^,^^25^ frequency,^26^ and quantile.^27^ These studies focused on a range of financial markets, such as stock markets,^24^ oil markets,^25^ and oil-stock markets.^26^^,^^27^ The results demonstrated that the spillovers across markets were heterogeneous across different sample periods. Furthermore, spillovers were more prominent during turmoil or crisis episodes, especially the global financial crisis and the COVID-19 pandemic.

To examine the time-varying structure of spillovers among financial time series, we establish a spillover network $G_{m}$ from the sub-time series $W_{m}$,^49^ where the window width denotes the length of the short-term period. The node of the network represents a time series in $W_{m}$, and the weighted edge represents the direction and magnitude of the spillovers between two series in the short-term period. Then, we obtain a sequence of spillover networks ${\left(G_{1},G_{2},\ldots ,G_{m}\right)}_{m=1}^{M}$. The spillovers between series i and j in network $G_{m}$ are calculated using the GARCH-BEKK (1,1) of Robert F Engle and Kroner.^54^ The model consists of a conditional mean equation and a conditional variance equation, which can be denoted as follows.

The conditional mean equation:

(Equation 9) $$D_{t}\left(m\right)=\left[\begin{array}{c} D_{i,t}\left(m\right)\\ D_{j,t}\left(m\right) \end{array}\right]=\left[\begin{array}{c} \phi_{m}^{i}\\ \phi_{m}^{j} \end{array}\right]+\left[\begin{array}{cc} \phi_{11} & \phi_{12}\\ \phi_{21} & \phi_{22} \end{array}\right]\left[\begin{array}{c} D_{i,t-1}\left(m\right)\\ D_{j,t-1}\left(m\right) \end{array}\right]+\epsilon_{t}\left(m\right)$$

where ${\;D}_{t}\left(m\right)$ is a vector for series i and series j at time t in the sub-time series $W_{m}$. $\phi_{m}^{i}$, and $\phi_{m}^{j}$ represent the long-term drift coefficients for series i and series j, respectively. $\epsilon_{t}\left(m\right)$ represents the random errors at time t, where $\epsilon_{t}\left(m\right)|\Omega_{t-1}∼N\left\{0,H_{t}\left(m\right)\right\}$.

The conditional variance equation:

(Equation 10) $$H_{t}\left(m\right)=C^{\prime }C+A^{\prime }\epsilon_{t-1}\left(m\right)\epsilon_{t-1}^{\prime }\left(m\right)A+B^{\prime }H_{t-1}\left(m\right)B$$

(Equation 11) $$A=\left[\begin{array}{cc} a_{11} & a_{12}\\ a_{21} & a_{22} \end{array}\right],B=\left[\begin{array}{cc} b_{11} & b_{12}\\ b_{21} & b_{22} \end{array}\right]\;and\;C=\left[\begin{array}{cc} c_{11} & 0\\ c_{21} & c_{22} \end{array}\right]$$

where A represents the conditional residual matrix, B represents the coefficient of the conditional covariance matrix, and C is the constant coefficient matrix. Furthermore, ${Sp}_{i,j}\left(m\right)$ represents the spillover from series i to series j in the sub-time series $W_{m}$; ${Sp}_{j,i}\left(m\right)$ is the spillover from series j to series i in the sub-time series $W_{m}$. They can be defined as follows:

(Equation 12) $${Sp}_{i,j}\left(m\right)=\left|a_{12}\right|+\left|b_{12}\right|\;and\;{Sp}_{j,i}\left(m\right)=\left|a_{21}\right|+\left|b_{21}\right|$$

Next, we investigate the dynamic topological structure of spillover networks to reveal a market’s return volatility feature in detail.^25^^,^^55^ The indicators of a network $G_{m}$ are denoted as $I_{m}=\left[\text{Diam}\left(G_{m}\right)\;\text{APL}\left(G_{m}\right)\;\text{Dens}\left(G_{m}\right)\;\text{AOS}\left(G_{m}\right)\;\text{ABC}\left(G_{m}\right)\;\text{ACC}\left(G_{m}\right)\right]$. The longest of all the calculated shortest paths in a spillover network $G_{m}$ is measured by the network diameter $\text{Diam}\left(G_{m}\right)$, which reflects the linear size of a spillover network. The average number of steps along the shortest paths for all possible pairs of network nodes is calculated by the average path length $\text{APL}\left(G_{m}\right)$, which reflects the transmission performance among the spillover flows. The network density $\text{Dens}\left(G_{m}\right)$ is defined as the ratio of the number of edges to the number of possible edges in a spillover network. The out-strength of a node is the magnitude of all spillovers from the node to others, and the average out-strength $\text{AOS}\left(G_{m}\right)$ reflects the average value of the out-strength of the nodes, which indicates the average importance of the spillover flows in a spillover network. The degree of the node as the bridge of the links in a spillover network is measured by its betweenness centrality, and the average betweenness centrality $\text{ABC}\left(G_{m}\right)$ is the mean value of the betweenness centrality of the nodes. The closeness centrality of a node measures its average distance (inverse distance) to other nodes, and the average closeness centrality $\text{ACC}\left(G_{m}\right)$ represents the average value of the closeness centralities of the nodes.

Based on the above method, we can obtain a sequence of spillover networks from a windowed time series, where the spillover networks evolve into each other. A network indicator matrix, which reflects the time-varying structure of spillovers Index(W), can be denoted as:

(Equation 13) $$\left[\begin{array}{c} \begin{array}{cc} \text{Diam}\left(G_{1}\right)\;\text{APL}\left(G_{1}\right) & \text{Dens}\left(G_{1}\right)\; \end{array}\text{AOS}\left(G_{1}\right)\;\text{ABC}\left(G_{1}\right)\;\text{ACC}\left(G_{1}\right)\\ \begin{array}{c} \ldots \\ \begin{array}{c} \begin{array}{c} \begin{array}{cc} \text{Diam}\left(G_{m}\right)\;\text{APL}\left(G_{m}\right) & \text{Dens}\left(G_{m}\right)\; \end{array}\text{AOS}\left(G_{m}\right)\;\text{ABC}\left(G_{m}\right)\;\text{ACC}\left(G_{m}\right)\\ \ldots \end{array}\\ \begin{array}{cc} \text{Diam}\left(G_{M}\right)\;\text{APL}\left(G_{M}\right) & \text{Dens}\left(G_{M}\right)\; \end{array}\text{AOS}\left(G_{M}\right)\;\text{ABC}\left(G_{M}\right)\;\text{ACC}\left(G_{M}\right) \end{array} \end{array} \end{array}\right]$$

#### Early warning model via machine learning

##### Define the early warning signals

The days before regime switching occurs are referred to as early warning days in this section because they are related to the early warning signals of regime switching. It draws inspiration from the Sevim et al.^56^ of a perfect signal. If regime switching is expected to occur with the upcoming γ early warning days, the early warning signal is represented by a value of 1, and if not, it is represented by a value of 0. The early warning signal function can be defined as follows:

(Equation 14) $${SP}_{m}=\left\{ \begin{array}{c} \;1,\text{if}\;\exists \;k=1,2,\ldots ,\gamma \;s\text{ubject}\;\text{to}\;R_{m+k-1}\neq R_{m+k}\\ 0,\text{otherwise} \end{array}\right.$$

An example of early warning signals can be given in Table S1 in the supplemental information. Based on this definition, the early warning signals of regime switching can be described as $SP={\left({SP}_{1},{SP}_{2},\ldots ,{SP}_{m}\right)}_{m=1}^{M}$.

The following is a description of the early warning model of regime switching:

Step a1: Define the early warning signals SP as the output variable and the corresponding time-varying structures of spillover networks Index(W) as the input variable.

Step a2: Use a machine learning model to investigate the nonlinear relationship between the output variable and input variable, which is defined as Noli(Index(W))=SP.

Step a3: Measure the model performance and calculate the early warning signals.

##### Machine learning model

A machine learning model^57^^,^^58^ provides a useful framework for examining structural changes in data and predicting future trends in time series. The primary idea is to predict unseen data using the topological structure of the reconstructed complex network from the original time series.^59^^,^^60^ These models have been successfully applied in various fields, such as the financial market,^61^^,^^62^ neuroscience,^63^ and climate.^64^ To address the nonlinear relationship between early warning signals and the time-varying structure of the spillover network, this paper introduces six typical machine learning models, including SVM, GBDT, ANN, DNN, RF and KNN, to investigate the early warning of regime switching. The main characteristics of these models can be given in Table S2 in the supplemental information.

### Quantification or statistical analyses

To compare the performance of machine learning models, three performance criteria can be used as follows:

(Equation 15) Precision=TP/(TP+FP)

(Equation 16) Sensitivity=TP/(TP+FN)

(Equation 17) F-measure=2∗Precision∗Sensitivity/(Precision+Sensitivity)

(Equation 18) Accuracy=(TP+TN)/(TP+TN+FP+FN)

where TP, TN, FP, and FN represent true positive, true negative, false positive, and false negative, respectively. A confusion matrix including TP, TN, FP and FN is shown in Table S3 in the supplemental information. Accuracy measures the proportion of correctly classified samples. Precision is the ratio of true positives to all positives. Sensitivity (or recall) is a measure of a model that correctly identifies true positives. Precision and sensitivity sometimes appear in contradictory situations, indicating that the sensitivity is lower when the precision is greater. The F-measure (or F-score) is an indicator that integrates precision and sensitivity and can be used in model competition. In addition, the performance of a model depends on the research question and database being used.

### Additional resources

There are no additional resources to include in this study.
