# Supplementary material for: Early warning of regime switching in a complex financial system from a spillover network dynamic perspective
Source: iScience. 2025 Jan 30;28(3):111924. doi: 10.1016/j.isci.2025.111924 (PMC11976486; doi:10.1016/j.isci.2025.111924)
Supplement: Document S1. Figures S1–S13 and Tables S1–S3 [file mmc1.pdf]

iScience, Volume *28*

Supplemental information

Early warning of regime switching in a complex financial system from a spillover network dynamic perspective

Sufang An, Xiangyun Gao, Feng An, and Tao Wu

Figures and tables in method details

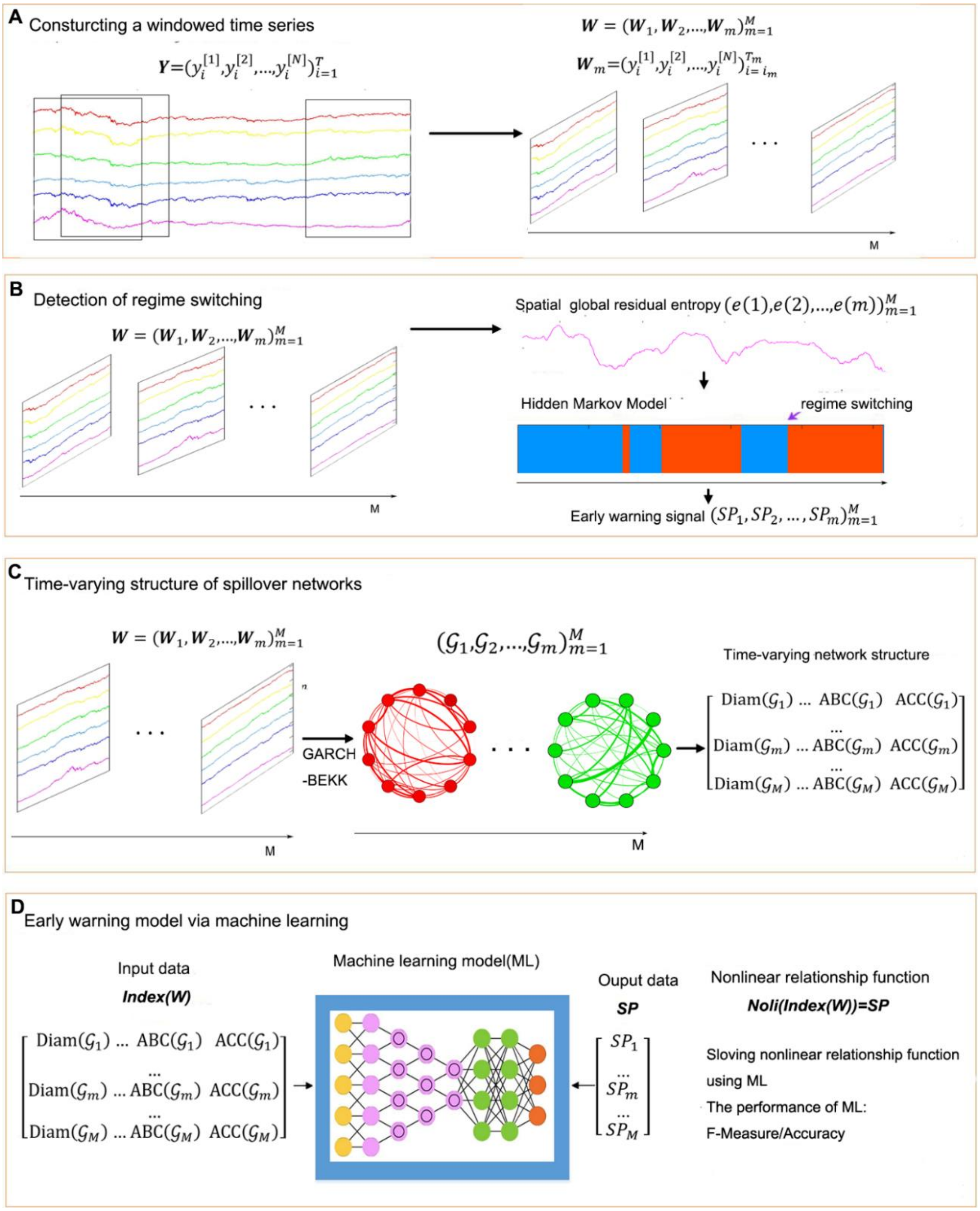

Figure S1. The process of the SN-ML model, related to STAR Methods

(A) Constructing a window time series.

(B) Detection of regime switching.

(C) Time-varying structure of spillover networks.

(D) Early warning model via machine learning.

Table S1. An example of early warning signals, related to STAR Methods

Day	State	Regime Switching	Early Warning Signal
0	Low	0	0
1	Low	0	1
2	Low	0	1
3	Low	0	1
4	Low	0	1
5	Low	0	1
6	High	1	0
7	High	0	0

Notes: Regime switching takes a value of 1 if the state of the day is different from that of the previous day. On Day 0, the original value of regime switching =0, and the original value of the early warning signal=0. It indicates that the early warning signal series always equals a value of 1 before five days if regime switching occurs on Day 6.

Table S2. Six typical machine learning models, related to STAR Methods

Model	Main Advantages	Main Disadvantages	Applied Fields
SVM	Its basic model is a linear classifier with the largest margin defined as the feature space. It is a nonlinear classifier due to its kernel functions; It has a good generalization ability for a binary classification.	It is difficult to deal with a great number of sample data and the multi classifications. It is sensitive to parameters.	Finance[S1], power [S2]and information fields [S3]
GBDT	It is an ensemble of decision trees, in which it combines several decision trees as the prediction model; it is a well-known binary classification model with a great generalization ability	It takes a long time to train the model. It is sensitive to the specific values and it is need to adjust the parameters of the model.	Environment [S4] and geography [S5].
ANN	ANNs include numerous models, such as the back-propagation algorithm [S6] and RPROP algorithm [S7]. Their essence reflects the behaviour of the human brain and simulates the way in which neurons signal to one another. It is easy to deal with train high-dimensional data and nonlinear data.	It takes a long time and lots of hardware resources to train the model.	Marking manaagement[S8] and eenvironmental science[S9]
DNN	DNNs are a type of deep learning architecture based on an ANN. The DNNs are typical feedforward networks with multiple layers between input and output layers.	It limits lots of parameters which may lead to overfitting. It requires a large amount of computation, long training time and high hardware resources.	Speech recognition [S10],quantum science[S11], and brain science [S12].

RF	It is an ensemble learning algorithm that consists of many decision trees. It can be used to train high-dimensional data, and the process can be used in the Parrel method. It works well for unbalanced data and increases the accuracy of the decision tree model.	It is difficult to control the inner part of the RF because it resembles a black box, and it works poorly for low-dimensional data.	Finance[S13], medicine [S14], and earth science [S15].
KNN	It is a supervised nonlinear classification model. depends on the K values (neighbours). No assumptions were made regarding the data. The accuracy was high and insensitive to outliers.	The number of computations in the model was large. When the data are unbalanced, accuracy is low. Compared to decision trees, interpretability was low.	Medicine [S16] ,finance [S17] and energy [S18]

Table S3. Confusion matrix representation for two-class classification, related to STAR Methods

Confusion Matrix		Actual Classification	
		Positive	Negative
Model Classification	Positive	TP	FP
	Negative	FN	TN

Note: The TP, TN, FP, and FN in this table represent true positive, true negative, false positive, and false negative, respectively.

Figures in robustness analysis

(1) Figures in original database using a window width of 200

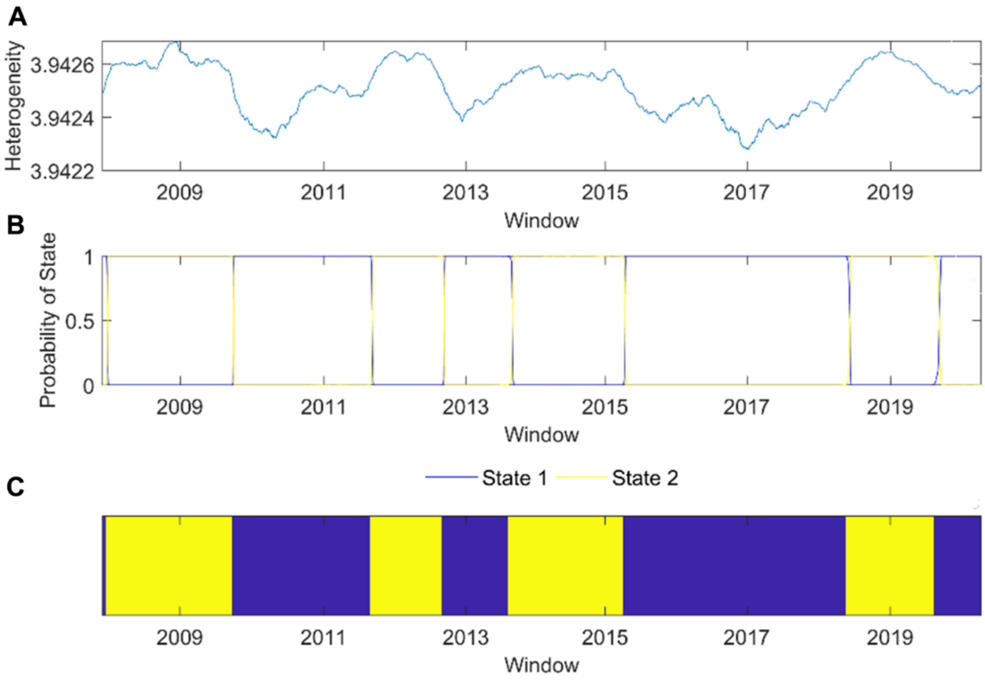

Figure S2. Detection of regime switching in a complex financial system under a window width of 200, related to Robustness analysis

(A) Spatial global residual entropy of the windowed time series. The window is the last day of the sub time series in the windowed time series.

(B) Probability of two states.

(C) The states of the windowed time series. The yellow and blue parts reflect the high and low states, respectively. The regime switching represents the switch between the high state and the low state.

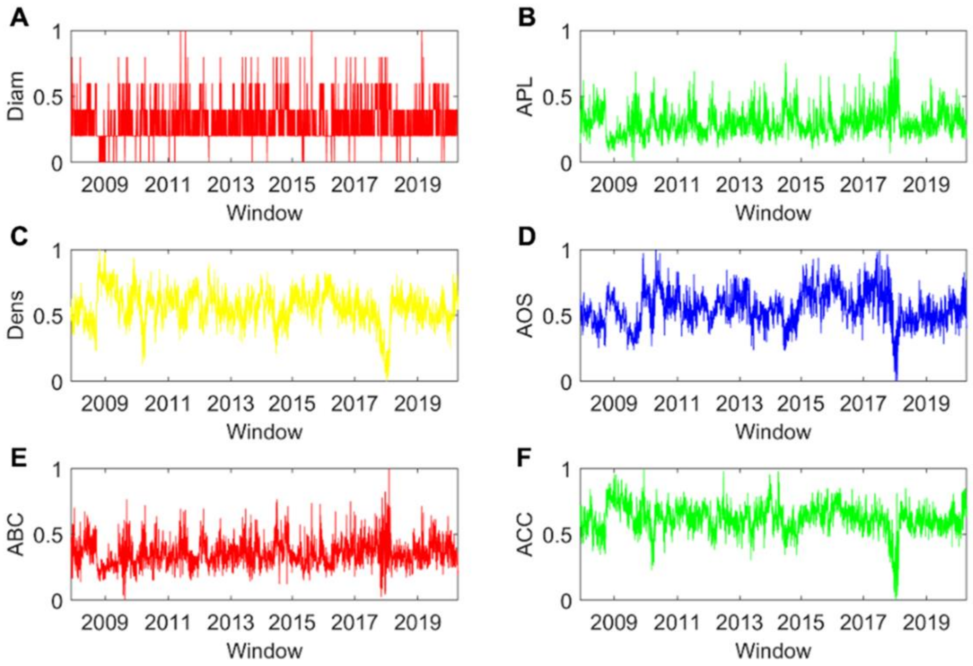

Figure S3. Dynamic topological structure of the spillover network under a window width of 200, related to Robustness analysis

The results are normalized network indicators.

(A) Dynamic diameter of the spillover network. Diam represents diameter.

(B) Dynamic APL of the spillover network. APL represents average path length.

(C) Dynamic density of the spillover network. Dens represents density.

(D) Dynamic AOS of the spillover network. AOS represents average out-strength

(E) Dynamic ABC of the spillover network. ABC represents average betweenness centrality

(F) Dynamic ACC of the spillover network. ACC represents average closeness centrality.

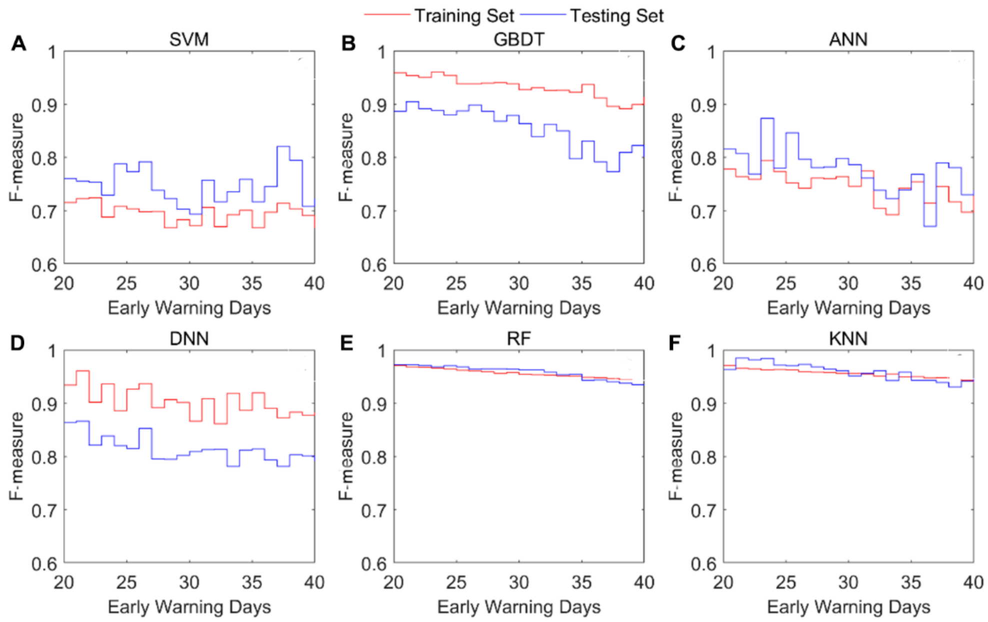

Figure S4. Dynamic F-measures of the training set and testing set in the machine learning models under a window width of 200, related to Robustness analysis

(A) Dynamic F-measures of the training set and testing set in the SVM.

(B) Dynamic F-measures of the training set and testing set in the GBDT.

(C) Dynamic F-measures of the training set and testing set in the ANN.

(D) Dynamic F-measures of the training set and testing set in the DNN.

(E) Dynamic F-measures of the training set and testing set in the RF.

(F) Dynamic F-measures of the training set and testing set in the KNN.

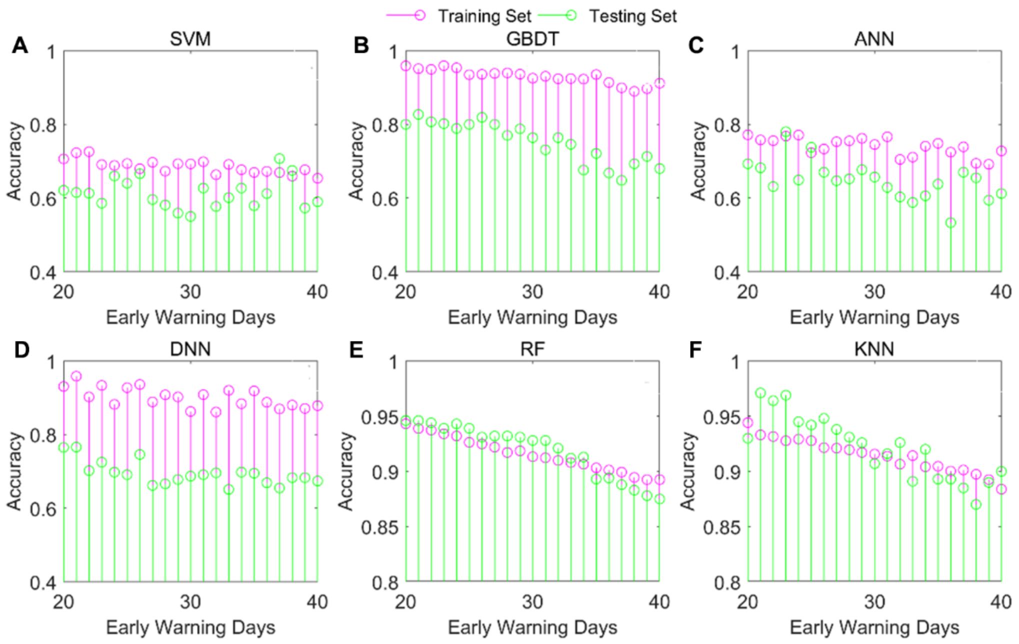

Figure S5. Dynamic accuracies of the training set and testing set in the machine learning models under a window width of 200, related to Robustness analysis

(A) Dynamic accuracies of the training set and testing set in the SVM.

(B) Dynamic accuracies of the training set and testing set in the GBDT.

(C) Dynamic accuracies of the training set and testing set in the ANN.

(D) Dynamic accuracies of the training set and testing set in the DNN.

(E) Dynamic accuracies of the training set and testing set in the RF.

(F) Dynamic accuracies of the training set and testing set in the KNN.

(2) Figures in a new daily database

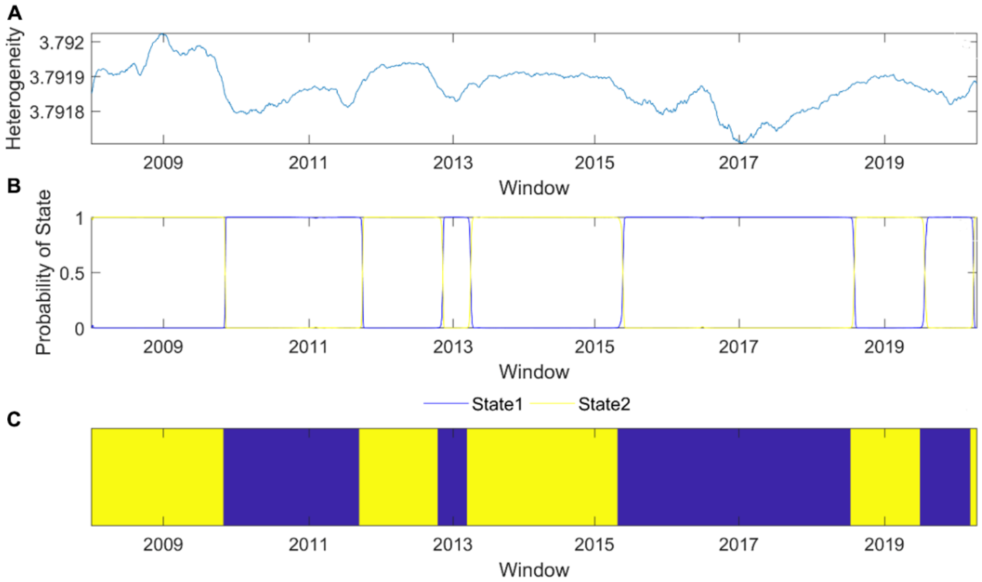

Figure S6. Detection of regime switching in a complex financial system based on a new daily database, related to Robustness analysis

(A) Spatial global residual entropy of the windowed time series. The window is the last day of the sub time series in the windowed time series.

(B) Probability of two states.

(C) The states of the windowed time series. The yellow and blue parts reflect the high and low states, respectively. The regime switching represents the switch between the high state and the low state.

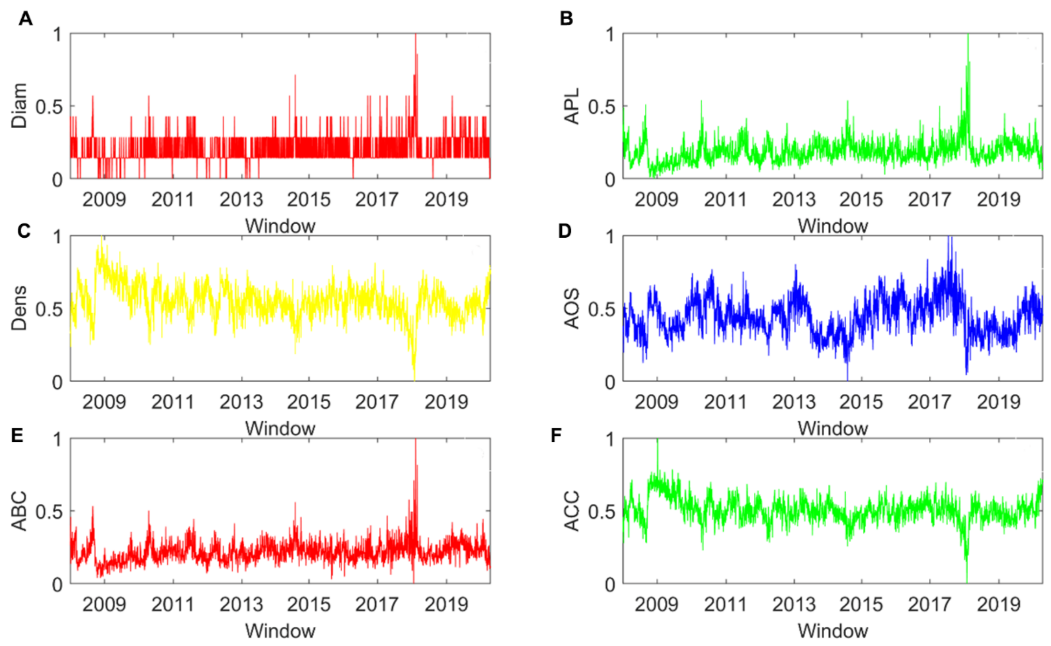

Figure S7. Dynamic topological structure of the spillover network based on a new daily database, related to Robustness analysis

The results are normalized network indicators.

(A) Dynamic diameter of the spillover network. Diam represents diameter.

(B) Dynamic APL of the spillover network. APL represents average path length.

(C) Dynamic density of the spillover network. Dens represents density.

(D) Dynamic AOS of the spillover network. AOS represents average out-strength

(E) Dynamic ABC of the spillover network. ABC represents average betweenness centrality

(F) Dynamic ACC of the spillover network. ACC represents average closeness centrality.

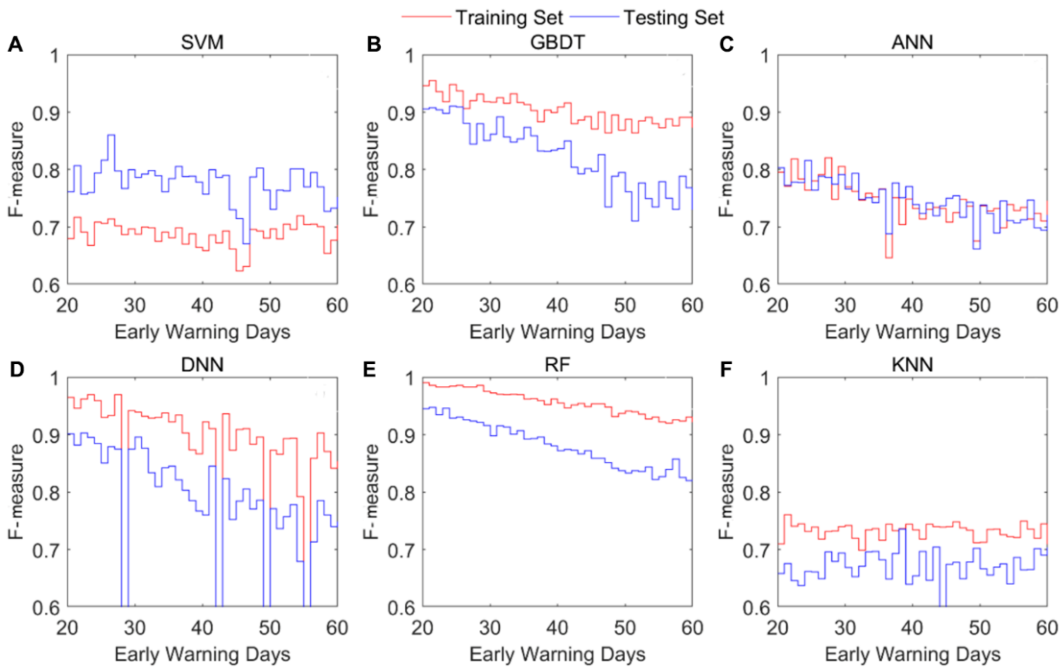

Figure S8. Dynamic F-measures of the training set and testing set in the machine learning models based on a new daily database, related to Robustness analysis

We randomly select 70% of the database as the training set and 30% of the database as the testing set.

(A) Dynamic F-measures of the training set and testing set in the SVM.

(B) Dynamic F-measures of the training set and testing set in the GBDT.

(C) Dynamic F-measures of the training set and testing set in the ANN.

(D) Dynamic F-measures of the training set and testing set in the DNN.

(E) Dynamic F-measures of the training set and testing set in the RF.

(F) Dynamic F-measures of the training set and testing set in the KNN.

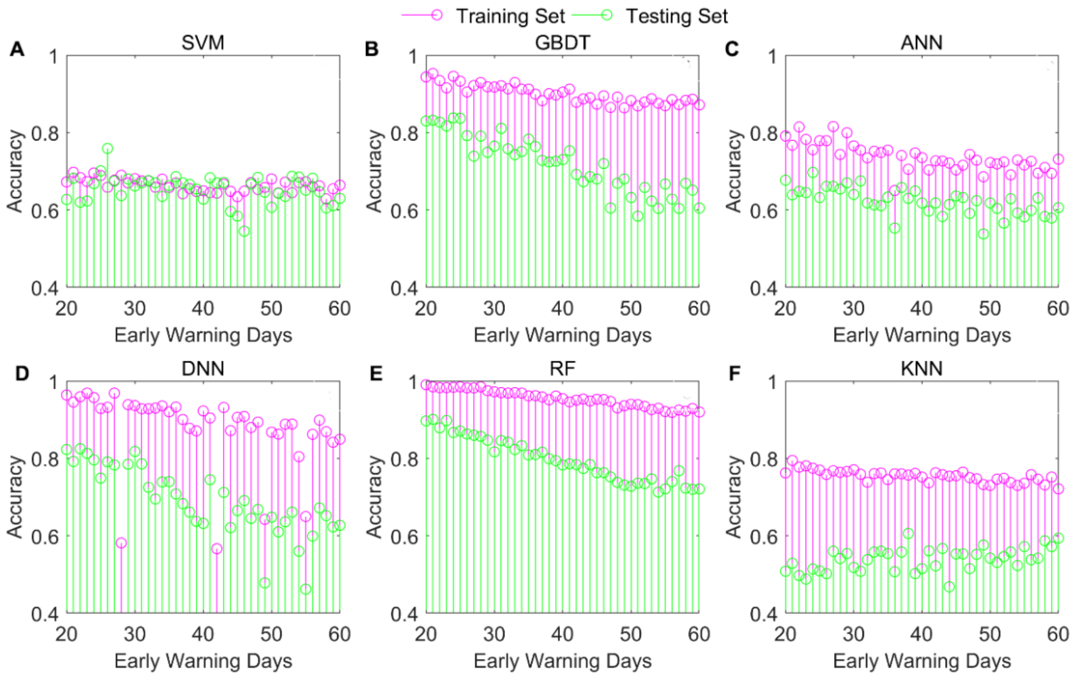

Figure S9. Dynamic accuracies of the training set and testing set in the machine learning models based on a new daily database, related to Robustness analysis

(A) Dynamic accuracies of the training set and testing set in the SVM.

(B) Dynamic accuracies of the training set and testing set in the GBDT.

(C) Dynamic accuracies of the training set and testing set in the ANN.

(D) Dynamic accuracies of the training set and testing set in the DNN.

(E) Dynamic accuracies of the training set and testing set in the RF.

(F) Dynamic accuracies of the training set and testing set in the KNN.

(3) Figures in a weekly database

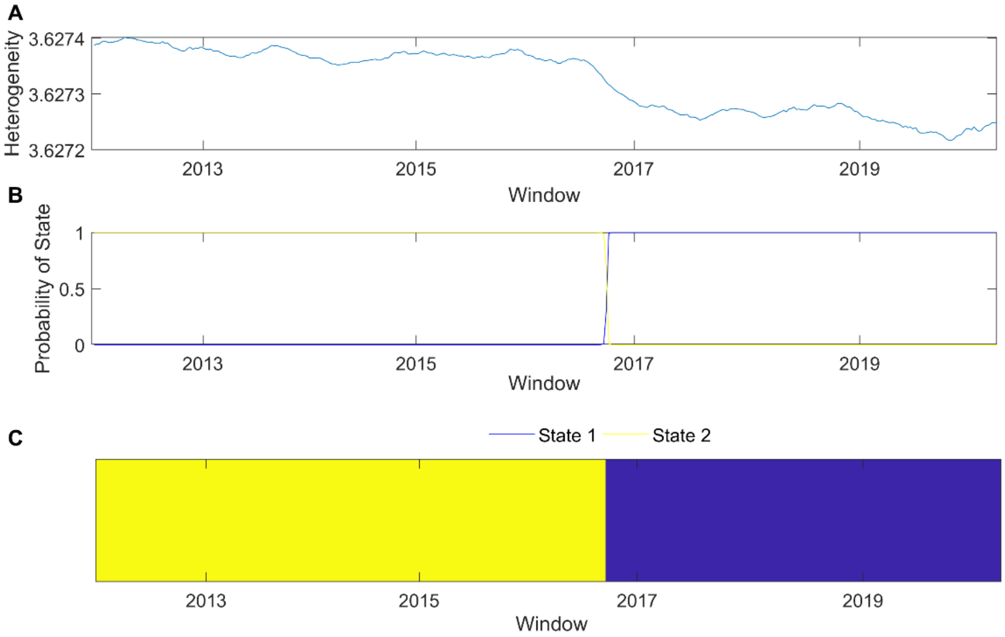

Figure S10. Detection of regime switching in a complex financial system based on a weekly database, related to Robustness analysis

(A) Spatial global residual entropy of the windowed time series. The window is the last day of the sub time series in the windowed time series.

(B) Probability of two states.

(C) The states of the windowed time series. The yellow and blue parts reflect the high and low states, respectively. The regime switching represents the switch between the high state and the low state.

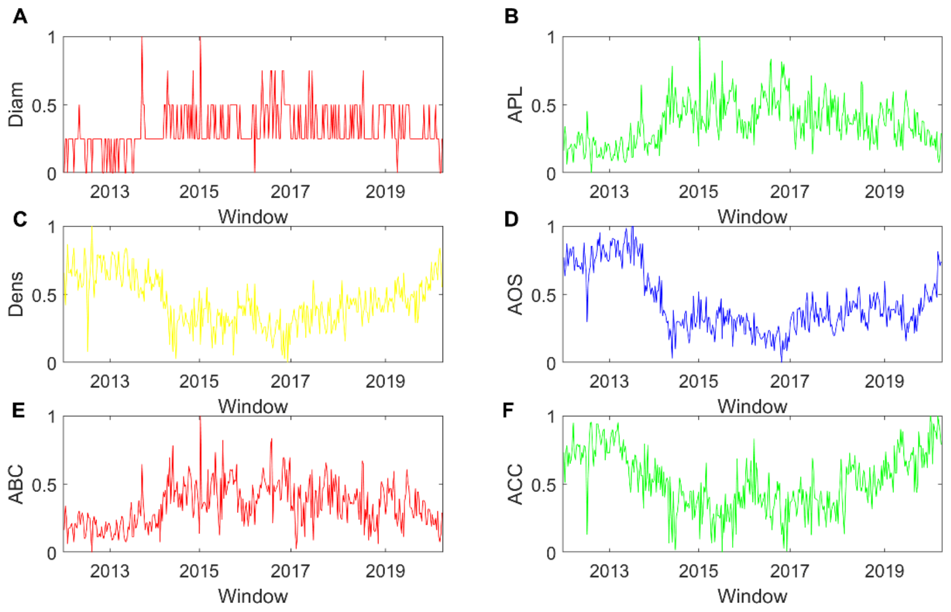

Figure S11. Dynamic topological structure of the spillover network based on a weekly database, related to Robustness analysis

The results are normalized network indicators.

(A) Dynamic diameter of the spillover network. Diam represents diameter.

(B) Dynamic APL of the spillover network. APL represents average path length.

(C) Dynamic density of the spillover network. Dens represents density.

(D) Dynamic AOS of the spillover network. AOS represents average out-strength

(E) Dynamic ABC of the spillover network. ABC represents average betweenness centrality

(F) Dynamic ACC of the spillover network. ACC represents average closeness centrality.

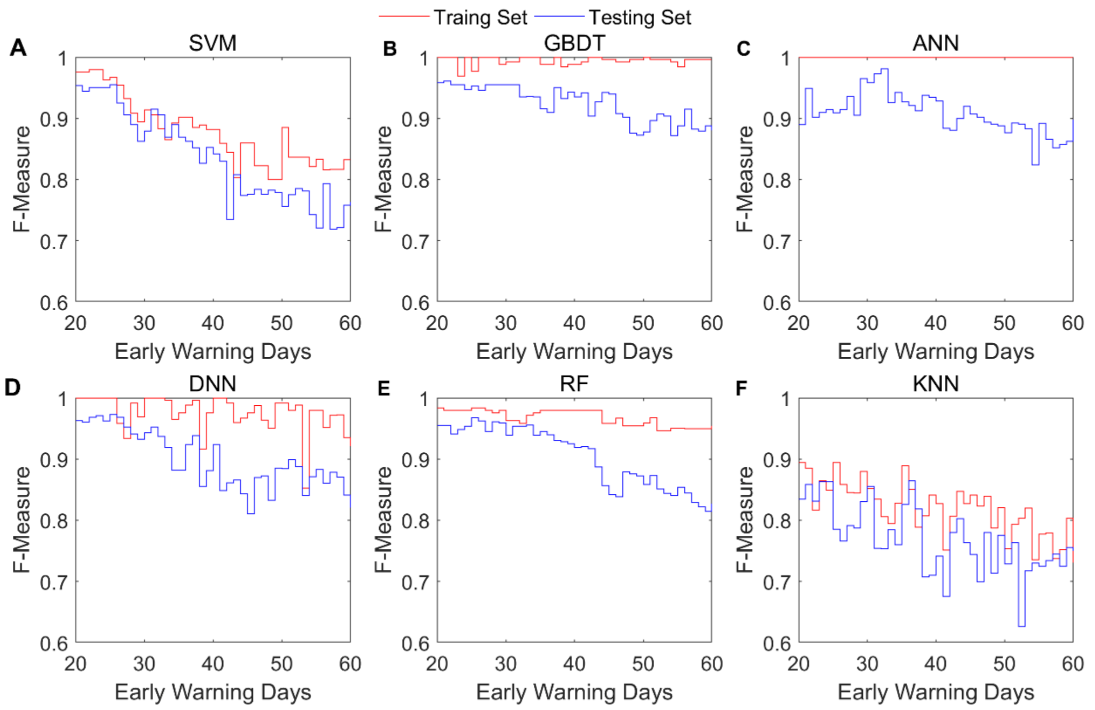

Figure S12. Dynamic F-measures of the training set and testing set in the machine learning models based on a weekly database, related to Robustness analysis

We randomly select 70% of the database as the training set and 30% of the database as the testing set.

(A) Dynamic F-measures of the training set and testing set in the SVM.

(B) Dynamic F-measures of the training set and testing set in the GBDT.

(C) Dynamic F-measures of the training set and testing set in the ANN.

(D) Dynamic F-measures of the training set and testing set in the DNN.

(E) Dynamic F-measures of the training set and testing set in the RF.

(F) Dynamic F-measures of the training set and testing set in the KNN.

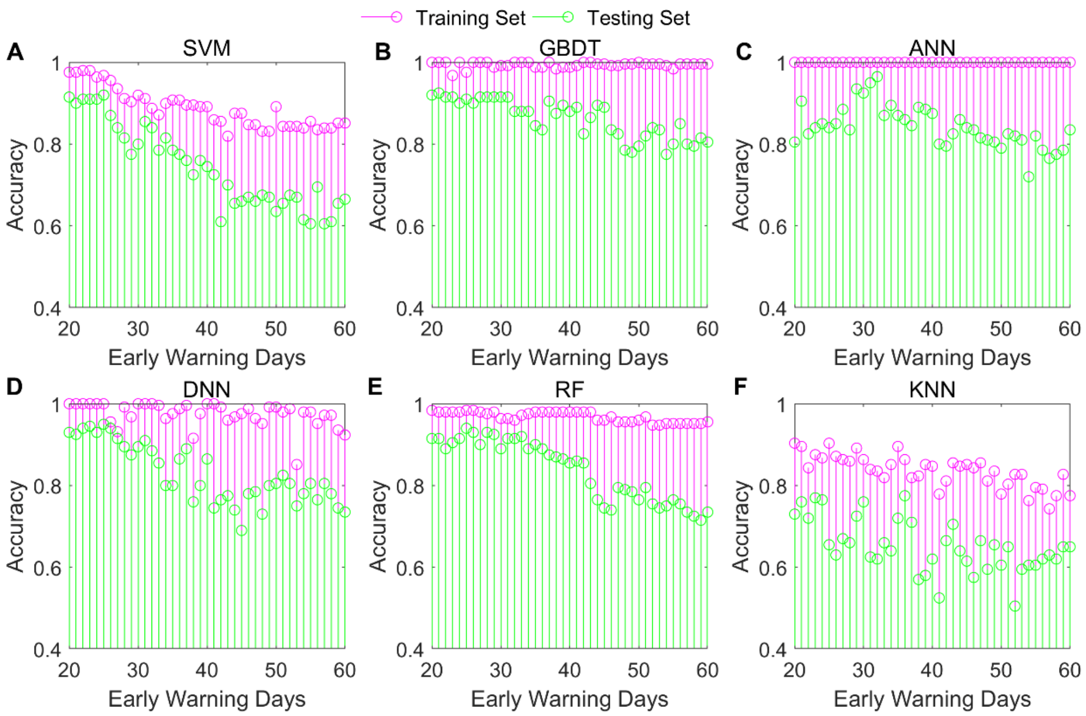

Figure S13. Dynamic accuracies of the training set and testing set in the machine learning models based on a weekly database, related to Robustness analysis

(A) Dynamic accuracies of the training set and testing set in the SVM.

(B) Dynamic accuracies of the training set and testing set in the GBDT.

(C) Dynamic accuracies of the training set and testing set in the ANN.

(D) Dynamic accuracies of the training set and testing set in the DNN.

(E) Dynamic accuracies of the training set and testing set in the RF.

(F) Dynamic accuracies of the training set and testing set in the KNN.

References

S1. Huang, X., and Guo, F. (2021). A kernel fuzzy twin SVM model for early warning systems of extreme financial risks. International Journal of Finance & Economics *26*, 1459-1468. https://doi.org/10.1002/ijfe.1858.

S2. Tran, V.-T., Nguyen, M.-K., Do, D.-T., and Vinnikov, D. (2021). An SVM scheme for three-level quasi-switched boost t-type Inverter with enhance voltage gain and capacitor voltage balance. IEEE Transactions on Power Electronics *36*, 11499-11508. https://doi.org/10.1109/TPEL.2021.3071011.

S3. Yuan, D., and Wang, X. (2024). Improved SVM Algorithm Financial Management Model for Data Mining. Journal of Information Knowledge Management *23*, 2450020. https://doi.org/10.1142/S0219649224500205.

S4. Cheng, N., Jing, D., Gu, Z., Cai, X., Shi, Z., Li, S., Chen, L., Li, W., and Wang, Q. (2024). Observation-based ozone formation rules by gradient boosting decision trees model in typical chemical industrial parks. Atmosphere *15*, 600. https://doi.org/10.3390/atmos15050600.

S5. Zhang, W., Lu, D., Liu, H., and Li, B. (2024). Varying built environment contexts and trip chain decisions: A multinomial-choice gradient boosting decision trees analysis. Travel Behaviour Society *34*, 100684. https://doi.org/10.1016/j.tbs.2023.100684.

S6. Rumelhart, D.E., Hinton, G.E., and Williams, R. (1986). Learning representations by back propagating errors Nature *323*, 533–536. http://doi.org/10.1038/323533a0.

S7. Riedmiller, M., and Braun, H. (1993). A direct adaptive method for faster backpropagation learning: the RPROP algorithm. Proceedings of the IEEE International Conference on Neural Networks (ICNN), 586-591. https://doi.org/10.1109/ICNN.1993.298623.

S8. Dang, T.-Q., Tan, G.W.-H., Aw, E.C.-X., Ooi, K.-B., Metri, B., and Dwivedi, Y.K. (2023). How to generate loyalty in mobile payment services? An integrative dual SEM-ANN analysis. International Journal of Bank Marketing *41*, 1177-1206. https://doi.org/10.1108/ijbm-05-2022-0202.

S9. Warren-Vega, W.M., Montes-Pena, K.D., Romero-Cano, L.A., and Zarate-Guzman, A. (2024). Development of an artificial neural network (ANN) for the prediction of a pilot scale mobile wastewater treatment plant performance. Journal of Environmental Management *366*, 121612. 10.1016/j.jenvman.2024.121612.

S10. Li, G., Liang, S., Nie, S., Liu, W., and Yang, Z. (2021). Deep neural network-based generalized sidelobe canceller for dual-channel far-field speech recognition. Neural Networks *141*, 225-237. https://doi.org/10.1016/j.neunet.2021.04.017.

S11. Sinha, P.K., and Marimuthu, R. (2024). Conglomeration of deep neural network and quantum learning for object detection: Status quo review. Knowledge-Based Systems *288*, 111480. https://doi.org/10.1016/j.knosys.2024.111480.

S12. Koide-Majima, N., Nishimoto, S., and Majima, K. (2024). Mental image reconstruction from human brain activity: Neural decoding of mental imagery via deep neural network-based Bayesian estimation. Neural Networks *170*, 349-363. https://doi.org/10.1016/j.neunet.2023.11.024.

S13. Sadorsky, P. (2021). A random forests approach to predicting clean energy stock prices. Journal of Risk Financial Management *14*, 48. https://doi.org/10.3390/jrfm14020048.

S14. Wang, J., Rao, C., Goh, M., and Xiao, X. (2023). Risk assessment of coronary heart disease based on cloud-random forest. Artificial Intelligence Review *56*, 203-232. https://doi.org/10.1007/s10462-022-10170-z.

S15. He, B., Armaghani, Jahed, D., and Lai, S.H. (2023). Assessment of tunnel blasting-induced overbreak: A novel metaheuristic-based random forest approach. Tunnelling Underground Space Technology *133*, 104979. https://doi.org/10.1016/j.tust.2022.104979.

S16. Uddin, S., Haque, I., Lu, H., Moni, M.A., and Gide, E. (2022). Comparative performance analysis of K-nearest neighbour (KNN) algorithm and its different variants for disease prediction. Scientific Reports *12*, 6256. https://doi.org/10.1038/s41598-022-10358-x.

S17. Rjoub, H., Adebayo, T.S., and Kirikkaleli, D. (2023). Blockchain technology-based FinTech banking sector involvement using adaptive neuro-fuzzy-based K-nearest neighbors algorithm. Financial Innovation *9*, 65. https://doi.org/10.1186/s40854-023-00469-3.

S18. Tang, Y., Chang, Y., and Li, K. (2023). Applications of K-nearest neighbor algorithm in intelligent diagnosis of wind turbine blades damage. Renewable Energy *212*, 855-864. https://doi.org/10.1016/j.renene.2023.05.087.
